# PGRMC1 Promotes Progestin-Dependent Proliferation of Breast Cancer Cells by Binding Prohibitins Resulting in Activation of ERα Signaling

**DOI:** 10.3390/cancers13225635

**Published:** 2021-11-11

**Authors:** Yingxue Bai, Marina Ludescher, Gereon Poschmann, Kai Stühler, Martine Wyrich, Julia Oles, André Franken, Mahdi Rivandi, Anna Abramova, Florian Reinhardt, Eugen Ruckhäberle, Dieter Niederacher, Tanja Fehm, Michael A. Cahill, Nadia Stamm, Hans Neubauer

**Affiliations:** 1 Department of Obstetrics and Gynecology, University Hospital and Medical Faculty of the Heinrich-Heine University Duesseldorf, Merowingerplatz 1a, 40225 Duesseldorf, Germany; yingxue.bai@med.uni-duesseldorf.de (Y.B.); ludescher.marina@gmail.com (M.L.); Martine.Wyrich@hhu.de (M.W.); julia.oles@hhu.de (J.O.); andre.franken@med.uni-duesseldorf.de (A.F.); mahdi.rivandi@med.uni-duesseldorf.de (M.R.); anna.abramova@med.uni-duesseldorf.de (A.A.); florian.reinhardt@med.uni-duesseldorf.de (F.R.); eugen.ruckhaeberle@med.uni-duesseldorf.de (E.R.); niederac@med.uni-duesseldorf.de (D.N.); tanja.fehm@med.uni-duesseldorf.de (T.F.); 2 Institute for Molecular Medicine, University Hospital and Medical Faculty of the Heinrich-Heine University Duesseldorf, Universitaetsstr. 1, 40225 Duesseldorf, Germany; gereon.poschmann@hhu.de (G.P.); kai.stuehler@hhu.de (K.S.); 3 Molecular Proteomics Laboratory, BMFZ, Heinrich Heine University Duesseldorf, Universitaetsstr. 1, 40225 Duesseldorf, Germany; 4 School of Dentistry and Medical Sciences, Charles Sturt University, Wagga Wagga, NSW 2678, Australia; mcahill@csu.edu.au; 5 ACRF Department of Cancer Biology and Therapeutics, The John Curtin School of Medical Research, Canberra, ACT 2601, Australia

**Keywords:** PGRMC1, progesterone, progestins, breast cancer, estrogen receptor, hormone therapy, PHB1, PHB2

## Abstract

**Simple Summary:**

Combined menopausal hormone therapy is associated with increased breast cancer risk in postmenopausal women. In our previous studies, progesterone receptor membrane component 1 (PGRMC1) was shown to play a role in progestins’ elicitation of enhanced proliferation of breast cancer cells. Here we describe a potential mechanism by which PGRMC1 contributes to breast cancer progression via interaction with prohibitins, inhibiting their function as transcriptional repressors. This facilitates estrogen receptor alpha (ERα) transcriptional activity and enhances oncogenic signaling upon treatment with certain progestins, including norethisterone and dydrogesterone. Our data underline the contribution of PGRMC1 to especially hormone receptor positive breast cancer pathogenesis and demonstrate the need for further studies to understand its role in cancer.

**Abstract:**

In previous studies, we reported that progesterone receptor membrane component 1 (PGRMC1) is implicated in progestin signaling and possibly associated with increased breast cancer risk upon combined hormone replacement therapy. To gain mechanistic insight, we searched for potential PGRMC1 interaction partners upon progestin treatment by co-immunoprecipitation and mass spectrometry. The interactions with the identified partners were further characterized with respect to PGRMC1 phosphorylation status and with emphasis on the crosstalk between PGRMC1 and estrogen receptor α (ERα). We report that PGRMC1 overexpression resulted in increased proliferation of hormone receptor positive breast cancer cell lines upon treatment with a subgroup of progestins including norethisterone and dydrogesterone that promote PGRMC1-phosphorylation on S181. The ERα modulators prohibitin-1 (PHB1) and prohibitin-2 (PHB2) interact with PGRMC1 in dependency on S181-phosphorylation upon treatment with the same progestins. Moreover, increased interaction between PGRMC1 and PHBs correlated with decreased binding of PHBs to ERα and subsequent ERα activation. Inhibition of either PGRMC1 or ERα abolished this effect. In summary, we provide strong evidence that activated PGRMC1 associates with PHBs, competitively removing them from ERα, which then can develop its transcriptional activities on target genes. This study emphasizes the role of PGRMC1 in a key breast cancer signaling pathway which may provide a new avenue to target hormone-dependent breast cancer.

## 1. Introduction

Breast cancer accounts for almost one in four cancer cases among women, making it the most commonly diagnosed cancer and the leading cause of cancer death (15.5%) [1]. Approximately 70% of all breast cancers diagnosed in postmenopausal women are hormone receptor-positive [2].

Factors contributing to breast cancer risk besides lifestyle are reproductive and hormonal risk factors like overall exposure to sex hormones during early menarche and late menopause, but also uptake of exogenous hormones like oral contraceptives and hormone replacement therapy (HT) [3,4,5]. The latter is administered peri- and post-menopausal for treatment of climacteric symptoms to improve quality of life [6]. HT that only includes the use of estrogens is referred to as estrogen-only hormone therapy (EHT) [7]. However, in addition to estrogens, HT usually includes co-treatment with progestins, synthetic derivates of gestagens, added to prevent the development of endometrial hyperplasia and an associated risk of endometrial cancer due to estrogen administration. This HT is referred to as combined estrogen-progestin hormone therapy (CHT) [8].

Various prospective large cohort studies, such as the Million Women Study (1,084,110 women) and the Women’s Health Initiative (27,547 women), overwhelmingly suggest that combined hormone therapy (CHT), relatively to EHT, increases the risk of breast cancer, indicating a potential role of progestins in breast carcinogenesis [7,9,10]. Fournier et al. demonstrated that the risk of breast cancer differs, depending on the type of progestin used. With a relative risk of 2.74 (95% confidence interval (CI): 1.42–5.29), 2.57 (95% CI: 1.81–3.65) and 2.11 (95% CI: 1.56–2.86) the progestins medrogestone, cyproterone acetate and norethisterone acetate were found to exhibit the highest breast cancer risk. In contrast to combined estrogen/progestin therapy, no increased breast cancer risk has been reported for the combined therapy of estrogens and progesterone (4-Pregnene-3,20-dione: hereafter P4) (relative risk: 1.08 (95% CI: 0.89–1.31) (relative risk: 1.08 (95% CI: 0.89–1.31) [6]. Although evidence points towards a significant contribution of certain progestins to breast cancer risk, the cellular mechanisms underlying this observation are unclear. Most effects observed upon progestin treatment refer to their action on the nuclear Progesterone Receptor (PR), but also other hormone receptors like the androgen receptor and the glucocorticoid receptor are reported to be targeted by progestins or their metabolites [11,12,13,14]. Recent studies further indicate potential effects of progestins on Progesterone Receptor Membrane Component-1 (PGRMC1) [15,16,17].

PGRMC1 is expressed in different cellular systems and contexts and has a wide range of cellular functions [17,18]. It was discovered by Meyer et al., when searching for alternative membranous high affinity P4 binding sites and was therefore suggested as a putative progesterone receptor [19,20]. Since then, PGRMC1 has been associated with P4 responses in various cell systems [21,22,23]. Furthermore, the multiple functions exerted by PGRMC1 include cholesterogenesis [24] and interactions with CYP450 enzymes that metabolize steroid hormones and chemotherapeutics [25,26].

In previous studies, we provided evidence that PGRMC1 is involved in the mode of action of progestins on breast cancer cells [27,28]. PGRMC1 was demonstrated to confer progestin responsiveness, which results in enhanced proliferation of MCF7 breast cancer cells in vitro and in vivo [27,29,30], indicating a potential role of PGRMC1 in increased breast cancer risk upon progestin-based HT [31]. We further examined the biological activity of progestins associated with regulation of PGRMC1 activity and discovered that PGRMC1 is phosphorylated at the Casein Kinase 2 (CK2) phosphorylation consensus site S181, and thus potentially activated by the progestin norethisterone (NET) [27]. Considering that PGRMC1 is expressed in breast tissue and overexpressed in breast cancer [16], further investigation of progestin-dependent PGRMC1 signaling in breast cancer cells is essential for a better understanding of the effects of progestins on breast cancer risk.

Therefore, the aim of this study was to gain deeper insight into PGRMC1-mediated breast cancer progression upon progestin treatment and the signaling pathways involved. For this purpose, potential PGRMC1-interaction partners in breast cancer cells were evaluated with norethisterone (acetate) (NET) treatment. A special focus was placed on progestin-dependent implication of PGRMC1 in ERα signaling and regulation of prohibitins (PHBs), which are reported to function as transcription factor modulators [32], and can occupy protein complexes with PGRMC1, although direct physical contact has not been demonstrated [33]. Here, we provide evidence that crosstalk exists between PGRMC1 and ERα that could promote progression of breast cancer.

## 2. Materials and Methods

### 2.1. Cells and Cell Culture

MCF7, T47D and MDA-MB-231 cells were purchased from the ATCC (Manassas, VA, USA (HTB-22, HTB-133 and CRM-HTB-26)). Cells overexpressing 3× human influenza hemagglutinin (HA)-tagged PGRMC1 (termed MCF7/PGRMC1, T47D/PGRMC1 and MDA-MB-231/PGRMC1) and their respective negative empty vector control cells (MCF7/EVC, T47D/EVC and MDA-MB-231/EVC) were generated via stable transfection with the expression vector pcDNA3.1/Hygro (+) (Thermo Fisher Scientific, Waltham, MA, USA, V87020), containing 3×HA-tagged PGRMC1 as described elsewhere [24,29]. Prior to this study, MCF7 cells overexpressing GFP-tagged PGRMC1 (termed MCF7/PGRMC1-GFP) [24] and the phosphorylation-deficient PGRMC1-site mutants S57A (MCF7/PGRMC1-S57A), S181A (MCF7/PGRMC1-S181A) and a double site mutant S57A/S181A (MCF7/PGRMC1-S57A/S181A) [34] were established and described in previous publications. PGRMC1-deficient MCF7 cells (MCF7/PGRMC1-KO) were generated by transient transfection of MCF7 cells with ‘PGRMC1 CRISPR/Cas9 KO Plasmid (h)’ (Santa Cruz Biotechnology, CA, USA, sc-401945, containing a mixture of three expression plasmids each encoding for the Cas9 enzyme and a PGRMC1-specific gRNA) using Lipofectamine 3000 (Thermo Fisher Scientific, L3000001). For control (MCF7/PGRMC1-KO/Control), cells were transfected with respective amount of ‘CRISPR/Cas9 KO Control Plasmid’ (Santa Cruz Biotechnology, sc-418922, encodes for the Cas9 enzyme and an unspecific gRNA). Cells were trypsinized 48 h post-transfection and single clones were selected by limiting dilution in 96-well Plates. Single cell colonies were screened for a successful PGRMC1-knockout by PCR, expanded and validated by western blotting and immunofluorescence staining. All cells were maintained in RPMI 1640 medium (Thermo Fisher Scientific, 2340229), supplemented with 10% (*v*/*v*) fetal bovine serum (Thermo Fisher Scientific, 2333352), 100 units/mL penicillin/streptomycin (Thermo Fisher Scientific, 2321118) and 0.025 mol/L HEPES (Thermo Fisher Scientific, 2192897) (hereafter referred to as complete medium) in a humidified incubator at 37 °C in the presence of 5% CO_2_. Cells (passage number ≤ 25) were regularly tested negative for mycoplasma and regularly authenticated by Microsynth AG (Balgach, Switzerland) using STR analysis.

### 2.2. Treatment

Hormones: For progestin treatment, cells were seeded in complete medium. After 24 h, the medium was changed to a phenol-red free RPMI 1640 medium (Thermo Fisher Scientific, 2300455) supplemented with 10% charcoal stripped fetal bovine serum (Thermo Fisher Scientific, 22361499), 100 units/mL penicillin/streptomycin (Thermo Fisher Scientific, 2321118) as well as 25 mM HEPES (hereafter referred to as stripped medium) and cells were incubated for another 48 h. Treatment was performed with NET (Sigma-Aldrich, St. Louis, MO, USA, N1200000), dydrogesterone (DYD) (Sigma-Aldrich, Y0001004), drospirenon (DSP) (Sigma-Aldrich, Y0001105), medroxyprogesterone (acetate) (MPA) (Sigma-Aldrich, M0250000), cyproterone (acetate) (CPA) (Sigma-Aldrich, C3283000), nomegestrel (acetate) (NOM) (Sigma-Aldrich, N1080005) and P4 (Sigma-Aldrich, Y0001665) at concentrations of 10^−6^, 10^−7^, 10^−8^ and 10^−9^ M (from 10^−2^ M stock solutions in DMSO) or the respective amount of DMSO (Sigma-Aldrich, D8418) as a control for a defined time period (72 h for MTT assay, 24 h for mass spectrometry analysis, RPPA, Western blot, co-immunoprecipitation (Co-IP), PLA and qRT-PCR) in stripped medium.

AG-205: To investigate effects of PGRMC1 inhibition on cell proliferation, MCF7 cells were treated with the putative PGRMC1 inhibitor AG-205 (Sigma-Aldrich, A1487) (see discussion). Cells were seeded in complete medium. After 24 h, the medium was changed to stripped medium (see above) with 25 × 10^−6^ M AG-205 (from 10^−2^ M stock solution in DMSO) or DMSO (0.25%) as control and incubated for another 48 h. Treatment was performed with NET or DYD at concentration of 10^−6^ M or DMSO (0.01%) as a control for 24 h in stripped medium.

Fulvestrant: To investigate effects of ERα downregulation on cell proliferation, MCF7/PGRMC1 and MCF7/EVC cells were treated with the selective estrogen receptor degrader fulvestrant (Sigma-Aldrich, Y0001399) [35]. Cells were seeded in complete medium containing 10^−7^ M fulvestrant (from 10^−3^ M stock solution) or DMSO (0.01%) as control. After 24 h, the medium was changed to stripped medium containing 10^−7^ M fulvestrant and cells were incubated for another 48 h. Treatment was performed with NET or DYD at concentrations of 10^−6^ M or DMSO (0.01%) as a control, for indicated time periods (72 h for MTT assay, 24 h for qRT-PCR) in stripped medium. For MTT assay, cells were cultured in fulvestrant-containing complete medium for 24 h before seeding into 96-well plates.

### 2.3. MTT Assay

Cells (1 × 10^4^ cells per well) were seeded in triplicates in 96-well plates in complete medium and grown for 24 h followed by starvation in stripped medium for 48 h and treatment with hormones as described above. On the day of the assay, cells were incubated with 0.25 mg/mL MTT solution (Sigma-Aldrich, 042K5313) for 3 h at 37 °C followed by 1 h of incubation with DMSO at 37 °C and 300 rpm in a microplate shaker. Absorption was measured at 540 nm using TECAN Spark^®^ spectrophotometer.

### 2.4. Western Blot Analysis

Cells were washed twice with ice cold PBS (Thermo Fisher Scientific, 2176323) and detached from the culture flasks using cell scrapers (Greiner Bio-One, Solingen, Germany). Cell pellets were lysed in RIPA buffer (50 mM TRIS (Sigma-Aldrich, T1503), 150 mM NaCl (VWR corporation, 16C030032), 1% NP-40 (Sigma-Aldrich, 74385), 0.5% sodium deoxycholate (Sigma-Aldrich, D6750), 0.1% SDS (Sigma-Aldrich, S34121136), phosphatase inhibitor (Roche, 49121300) and protease inhibitor (Roche, 49422800)) and protein concentration was determined using BCA assay (Thermo Fisher Scientific, 23225). 30 µg of whole cell protein supplemented with 4 × Laemmli buffer (Bio-Rad, Feldkirchen, Germany, 1610747) containing 2-Mercaptoethanol (Sigma-Aldrich, M6250) and the respective molecular weight marker were loaded onto Mini-PROTEAN^®^ Precast Gels (Bio-Rad, 4568123) and separated via SDS-Page at 100 V. Western blotting was performed as described elsewhere [27]. For signal detection the following antibodies were used: pSer181-PGRMC1 (EMBL, Monoclonal Antibody Core Facility, Monterotondo, Italy, #3G11A2, antibody not commercially available) [36], PGRMC1 (Cell signaling, Danvers, MA, USA, D6M5M), PHB1 (Cell signaling, 2426S), PHB2 (Cell signaling, 14085S) and β-actin (Santa Cruz Biotechnology, sc-47778). For validation of PGRMC1 knockout, we additionally used a second anti-PGRMC1 antibody (Abcam, Cambridge, UK, ab48012).

### 2.5. Co-Immunoprecipitation

Co-IP of HA-tagged PGRMC1 and HA-tagged PGRMC1-variants was performed as previously described [24].

### 2.6. Mass Spectrometry

PGRMC1 was immunoprecipitated from four individual replicates MCF7/PGRMC1 cells of following groups: HA-PGRMC1 DMSO, HA-PGRMC1 NET, GFP-PGRMC1 DMSO, GFP-PGRMC1 NET. Samples were processed by in-gel digestion and proteins were identified by mass spectrometry on an Orbitrap Elite instrument as described [27]. For data analysis, the MaxQuant environment (version 1.5.3.8, Max Planck Institute of Biochemistry, Planegg, Germany) was used with standard parameters if not otherwise stated. Spectra were searched against 20187 Swiss-Prot entries from the Homo sapiens proteome (UP000005640, downloaded on 18 November 2015 from UniProt KB). Label-free quantification was enabled as well as the ‘match between runs’ option. Tryptic cleavage specificity was chosen, as well as carbamidomethyl at cysteines as fixed and methionine oxidation, and acetylation at protein N-termini as variable modifications. Mass tolerances were 20 ppm (first search) and 4.5 ppm (second search after recalibration) for precursor masses and 0.5 Da for fragment masses. Peptides and proteins were accepted at a false discovery rate of 1%. Proteins were only considered for further analysis when showing at least two peptides and four valid values in at least one group. Missing values were imputed for global statistical calculation using random values from a downshifted normal distribution (1.8 SD downshift, width 0.3 SD). For statistical analysis, a two-way ANOVA was calculated within the R environment (The R foundation for statistical computing) and *p*-values were corrected for multiple testing by the method of Benjamini and Hochberg (corrected values are reported). Potential PGRMC1 interacting proteins were selected by a ratio HA/GFP > 2 and additionally a corrected *p*-value < 0.01 for the variable “TAG” in the ANOVA. For the 253 remaining proteins additionally Welch-tests were performed for the comparison of NET and DMSO treated HA-PGRMC1 samples. All captured proteins along with the statistical data are provided in Supplementary Table S1 (Appendix A). The mass spectrometry proteomics data have been deposited to the ProteomeXchange Consortium via the PRIDE [37] partner repository with the dataset identifier PXD028537.

### 2.7. qRT-PCR

RNA was isolated from a cell pellet of 1 × 10^6^ cells using the RNeasy Mini Kit (Qiagen, Hilden, Germany, 74104) according to the manufacturer’s specifications. Reverse transcription of RNA into cDNA was performed with the Omniscript RT kit (Qiagen, 205113) according to the manufacturer’s instructions. For qPCR, QuantiFast SYBR Green PCR kit (Qiagen, 204054) and RT² qPCR Primer assays for *TFF1* (Qiagen, PPH00998C) and *PDH* (Qiagen, PPH13220A) were used according to the manufacturer’s specifications. qPCR was performed using the LightCycler^®^ 480 System (Roche, Penzberg, Germany).

### 2.8. Immunofluorescence Staining

Cells were spun on glass slides, fixed with 4% PFA (Sigma-Aldrich, 20649296018) for 10 min at room temperature and washed three times for 5 min with washing buffer (Dako, Glostrup, Denmark, S3006). Afterwards, cells were permeabilized with 0.1% Triton X-100 (Sigma-Aldrich, T8787) in PBS for 10 min at room temperature (RT) and washed three times for 5 min with washing buffer. DAKO Protein Blocking Solution (Dako, X0909) was added and incubated for 1 h at RT. Cells were subjected to immunofluorescence staining with primary antibodies specific for PGRMC1 (Abcam ab48012), ERα (Abcam ab259427), PHB1 (Abcam ab75766) and PHB2 (Cell signaling 14085S) overnight at 4 °C. Afterwards, the slides were washed three times for 5 min with washing buffer and a respective fluorophore labeled secondary antibody (anti-goat: Invitrogen, A11055; anti-mouse: Invitrogen, 745480; anti-rabbit: Invitrogen, A31573) was added to the samples and incubated for 1 h at RT in the dark. The slides were washed three times for 5 min with washing buffer and incubated with DAPI (Thermo Fisher Scientific, 15733122) for 5 min at RT. Antibody incubation steps were performed in a humidified chamber. The slides were washed with distilled water, mounted with Fluorescent Mounting Medium (Dako, S3023) and dried overnight. The cells were examined by fluorescence microscopy using the Axioplan 2 Imaging fluorescence microscope.

### 2.9. Proximity Ligation Assay

The proximity ligation assay (PLA) procedure was performed using the Duolink^®^ PLA Kit (Sigma-Aldrich, DUO92008) according to the manufacturer’s instructions. Cells were spun on glass slides, fixed and permeabilized as described above. Incubation with the primary antibody cocktail containing anti-PGRMC1 antibody (Abcam, ab48012) or anti-ERα antibody (Abcam, ab259427) with PHB1 (Abcam, ab75766) or PHB2 (Cell signaling, 14085S) antibody was performed overnight at 4 °C. Negative control PLA was performed using respective isotype control antibodies (goat isotype, Abcam ab37373; mouse isotype, Abcam ab37355; rabbit isotype, Abcam ab37415). Nuclear DNA was labeled with DAPI for 10 min and slides examined by fluorescence microscopy within one week after storage at 4 °C in the dark. Each red dot represented a single interaction. Dots per cell were quantified using imageJ software [38].

### 2.10. Statistical Analysis

All experiments were performed as several independent biological replicates and repeated a minimum of three times. Results were reported as means with standard deviation. If not stated otherwise, data were tested for normal distribution using Shapiro-Wilk test and QQ normality plots and analyzed by one-way or two-way ANOVA (unmatched data) using GraphPad Prism (version 9.2.0, GraphPad Software, Inc., La Jolla, CA, USA). Differences between groups were calculated with the Bonferroni post-hoc test. *p* < 0.05 was considered as statistically significant. The exact test applied is described in the respective figure legend.

## 3. Results

### 3.1. PGRMC1 Promotes Proliferation of Breast Cancer Cells upon Progestin Treatment

**Figure 1:**
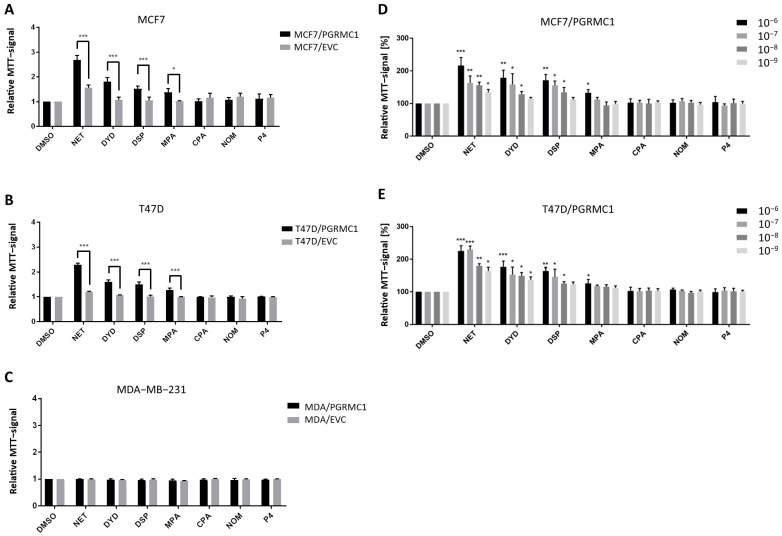
PGRMC1 promotes proliferation of breast cancer cells upon progestin treatment. Relative MTT-signal as surrogate for cell number of (**A**) MCF7/PGRMC1 and MCF7/EVC cells, (**B**) T47D/PGRMC1 and T47D/EVC cells, (**C**) MDA-MB-231/PGRMC1 and MDA-MB-231/EVC cells. Cells were treated with different progestins in the concentration of 10^−6^ M. Relative MTT-signal of (**D**) MCF7/PGRMC1 and (**E**) T47D/PGRMC1 cells treated with different concentrations of progestins (10^−6^–10^−9^ M) for 72 h. Values were normalized to respective DMSO treated cells. Statistical analysis was performed with twoway ANOVA and Bonferroni post−hoc test. *: *p* < 0.05, **: *p* < 0.01, ***: *p*< 0.001.

As already shown in previous studies, PGRMC1 represents a potential integration point and transmitter of progestin signals responsible for the growth and proliferation of breast cancer cells [27,29]. To further study this effect, we used the HA-tagged PGRMC1-overexpressing breast cancer cell lines MCF7/PGRMC1, T47D/PGRMC1 and MDA-MB-231/PGRMC1 as well as the respective empty vector control cell lines MCF7/EVC, T47D/EVC and MDA-MB-231/EVC, and performed the MTT assay to measure activated metabolism as surrogate for cell proliferation upon treatment with various progestins used in CHT. For the ERα/PR positive cell lines MCF7/PGRMC1 and T47D/PGRMC1, treatment with the progestins NET, DYD and DSP (10^−6^ M) significantly increased cell proliferation compared to the respective EVC cells while no effects were observed after treatment with CPA, NOM and P4 (10^−6^ M) (Figure 1A,B). For MCF7/PGRMC1 cells, significantly higher proliferation was also observed after treatment with MPA (10^−6^ M). In contrast, treatment of PGRMC1-overexpressing MDA-MB-231 cells with any progestin (10^−6^ M) or P4 (10^−6^ M) did not increase their proliferation compared to MDA-MB-231/EVC cells (Figure 1C), suggesting that progestin-mediated PGRMC1 signaling is mediated by proteins which are expressed in the HR positive cell lines MCF7 and T47D, but not in the triple-negative cell line MDA-MB-231.

To analyze if effects of progestins on MCF7/PGRMC1 and T47D/PGRMC1 cells can be observed at very low concentrations, cells were treated with progestins in concentrations ranging from 10^−6^ M to 10^−9^ M. For NET, we detected significantly higher proliferation for PGRMC1-overexpressing cells even at 10^−9^ M compared to DMSO treated cells (Figure 1D,E), whereas the respective EVC cells only responded at higher concentrations (Figure S1A,B). For DYD and DSP, significantly elevated proliferation compared to the DMSO control was observed at concentrations down to 10^−8^ M, while for MPA only the concentration of 10^−8^ M resulted in increased proliferation (Figure 1D,E). As in the previous experiment, CPA, NOM and P4 treatment did not influence proliferation of MCF7/PGRMC1 and T47D/PGRMC1 cells compared to DMSO control at any concentration (Figure 1D,E).

Taken together, these results demonstrate that PGRMC1-overexpression sensitizes ERα/PR positive luminal breast cancer cells to treatment with specific progestins (NET, DYD, DSP, and MPA; proliferation-promoting progestins, hereafter referred to as PPPs), while other progestins (CPA, NOM and P4; non-proliferation-promoting progestins, hereafter referred to as N-PPPs) did not enhance proliferation under any condition. The increase of proliferation in MCF7/EVC and T47D/EVC cells after treatment with NET, DYD and DSP or NET and DYD, respectively (Figure S1A,B), may be conducted via the endogenously expressed PGRMC1 which is still present in our system.

### 3.2. PGRMC1 Associates with the ERα-Modulators PHB1 and PHB2 upon Treatment with NET

**Figure 2:**
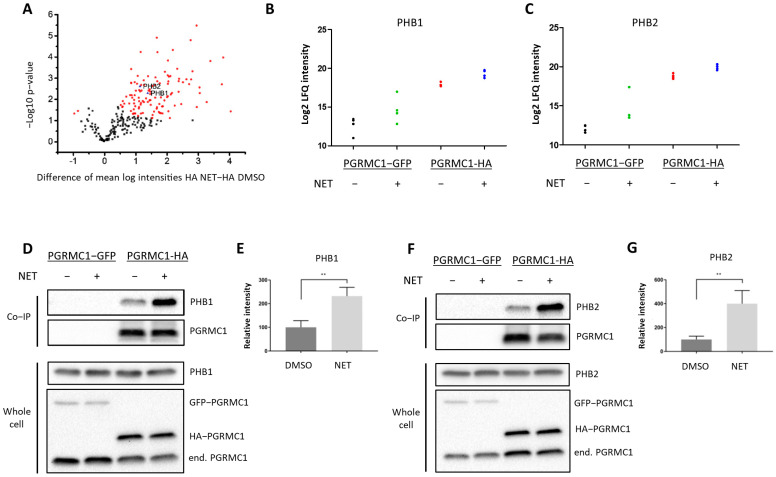
PGRMC1 interacts with the ERα-modulators PHB1 and PHB2 upon treatment with NET. Analysis of immunopurified (HAbased) samples of MCF7/PGRMC1-GFP cells (PGRMC1-GFP) and MCF7/PGRMC1 cells (PGRMC1-HA) treated with DMSO or NET (10^−6^ M) for co-precipitated proteins. (**A**) Volcano plot showing the result of a Welch’s *t*-test including 253 proteins with an increased abundance as revealed by a two-way ANOVA after HA-based enrichment. Proteins represented by red dots and blue triangles show a significantly altered abundance (FDR 0.01%). Mass spectrometry results for co-precipitated (**B**) PHB1 or (**C**) PHB2, log2 normalized intensity +: significantly different (Welch’s test). (**D**,**F**) Western blot analysis for co-precipitated (**D**) PHB1 or (**F**) PHB2 (upper panel) and the protein level of PHB1, PHB2 and PGRMC1 in whole cell lysates from the same cells (lower panel). (**E**,**G**) Densitometric analysis for precipitated (**E**) PHB1 or (**G**) PHB2. Signal intensity was normalized to PGRMC1-HA/DMSO. Difference between DMSO- and NET-treated samples was calculated with unpaired Student’s *t*-test. **: *p* < 0.01.

To gain insight into the mechanism by which PGRMC1 impacts proliferation of breast cancer cells, we screened for potential PGRMC1 interaction partners upon treatment with the PPP NET by mass spectrometry analysis of proteins co-immunoprecipitated from whole-cell lysates of MCF7/PGRMC1 cells utilizing an antibody directed against the HA-tag (Table S1). The volcano plot (Figure 2A) stratifies proteins exhibiting significantly increased signals in Co-IP pellets from the NET treated MCF7/PGRMC1 samples compared to Co-IP pellets from the corresponding DMSO treated MCF7/PGRMC1 cells. These could represent progestin-dependent PGRMC1 interaction partners. In the NET and the DMSO-treated MCF7/PGRMC1 cells, similar amounts of PGRMC1 proteins were precipitated as indicated by similar signal intensities (Figure S2). Significantly less PGRMC1 protein was precipitated by anti-HA beads in the DMSO and NET treated MCF7/PGRMC1-GFP control cells where PGRMC1 lacked the HA-tag (Figure S2), indicating the specificity of the assay for the presence of the HA-antigen in the PGRMC1 target protein.

Since the initial results point towards progestin-dependent increase of proliferation in ERα/PR-positive PGRMC1-overexpressing cell lines, we hypothesized that progestin-mediated PGRMC1 signaling is dependent on factors present in luminal cell types of breast cancer. Among proteins with higher signal intensities in NET treated MCF7/PGRMC1 cells compared to DMSO treated cells we found Prohibitin-1 (PHB1) (ANOVA *p*-value, corrected for multiple testing: tag 5.7 × 10^−7^, treatment 0.005) (Figure 2B) and Prohibitin-2 (PHB2) (ANOVA *p*-value, corrected for multiple testing: tag 3.2 × 10^−7^, treatment 0.01) (Figure 2C). We had previously identified that both proteins were present in AG-205-dependent Co-IP pellets with PGRMC1 [33]. Both PHBs are suggested to modulate transcriptional activity by directly or indirectly interacting with transcription factors, including transcriptional repression of ERα [39,40,41]. PHB2 is known as an ERα co-regulator that potentiates the inhibitory activities of antiestrogens and represses the activity of estrogens [42]. Due to their role as transcription factor modulators, we were interested in the association between PGRMC1 and PHB1 and PHB2 upon progestin treatment.

The results from mass spectrometry were verified by Co-IP followed by Western blot analysis. Both PHB1 (Figure 2D,E and Figure S13) and PHB2 (Figure 2F,G and Figure S14) exhibited significantly higher signals in MCF7/PGRMC1 cells after NET treatment. These results indicate augmented interaction of PHB1 and PHB2 with protein complexes containing PGRMC1 in the presence of NET compared to DMSO treatment.

### 3.3. PGRMC1-S181-Phosphorylation Correlates with Increased Cell Proliferation and PHB Binding upon Progestin Treatment

**Figure 3:**
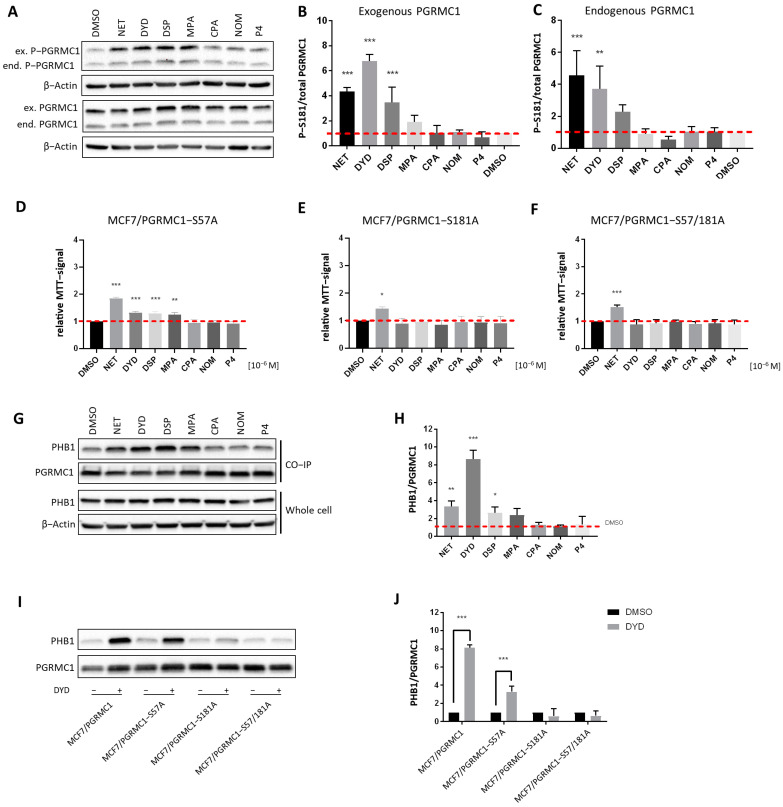
PGRMC1-S181-phosphorylation is essential for increased cell proliferation and PHB binding upon progestin treatment. (**A**) Western blot analysis of PGRMC1-S181-phosphorylation and PGRMC1 protein levels in whole cell lysates of MCF7/PGRMC1 cells after treatment with progestins (10^−6^ M) and DMSO. S181-phosphorylation occurs on both the endogenous PGRMC1 (lower band, ≈25 kDa) and exogenous HA-tagged PGRMC1 (upper band, ≈28 kDa). Densitometric analysis of Western blot results for S181-phosphorylation of (**B**) exogenous PGRMC1 and (**C**) endogenous PGRMC1 relatively to total PGRMC1 protein level. (**D**–**F**) Relative MTT signal as surrogate for cell number of (**D**) MCF7/PGRMC1-S57A, (**E**) MCF7/PGRMC1-S181A, (**F**) MCF7/PGRMC1-S57A/S181A cells treated with different progestins (all 10^−6^ M) or DMSO for 72 h. Values were normalized to DMSO treated cells. (**G**) Western blot analysis of immunopurified HA-tagged PGRMC1 and co-precipitated PHB1 from MCF7/PGRMC1 cells treated with different progestins (10^−6^ M) and DMSO (upper panel) and PHB1 protein level in whole cell lysates in the same cells (lower panel). (**H**) Densitometric analysis of co-precipitated PHB1 (**I**) Western blot analysis of immunopurified HA-tagged PGRMC1-variants and co-precipitated PHB1 after treatment with DYD (10^−6^ M) or DMSO. (**J**) Densitometric analysis of co-precipitated PHB1. (**B**,**C**,**H**,**J**) Signal intensity was normalized to corresponding DMSO-control and signal intensity of total PGRMC1 (**B**,**C**) or each precipitated PGRMC1-variant (**H**,**J**). Statistical analysis was performed by one-way ANOVA (**A**–**H**) or two-way ANOVA (**J**) and Bonferroni post-hoc tests. *: *p* < 0.05, **: *p* < 0.01, ***: *p* < 0.001.

PGRMC1 is subject to differential phosphorylation, which has been reported to potentially regulate its functions [43,44]. As previously published, we have investigated PGRMC1 phosphorylation upon progestin treatment in MCF7/PGRMC1 cells by Co-IP and subsequent mass spectrometry and identified S181 as the PGRMC1 site whose phosphorylation was significantly increased upon treatment with NET [27]. To further investigate a potential relationship between PGRMC1-S181 phosphorylation and elevated cell proliferation, we used MCF7/PGRMC1, MCF7/EVC and MCF7/PGRMC1-S181A cells, the latter of which express a S181A-phosphorylation deficient PGRMC1-variant. We measured PGRMC1-S181 phosphorylation by Western blot of whole cell lysates upon treatment with both PPPs as well as N-PPPs. Both endogenously and exogenously expressed PGRMC1 showed increased S181 phosphorylation in PPP-treated MCF7/PGRMC1 cells compared to DMSO-treated cells, whereas PGRMC1 protein levels were comparable for all cells (Figure 3A–C and Figure S15). For the exogenously expressed PGRMC1, the most prominent effect was observed after stimulation with NET, DYD and DSP, with a clear trend for MPA. MCF7/EVC cells also exhibited increased S181-phosphorylation of endogenous PGRMC1 upon PPP treatment (Figures S3A,B and S18). In MCF7/PGRMC1-S181A cells, similar results were observed, except that the exogenous PGRMC1-S181A protein was not phosphorylated on S181 (Figures S3C,D and S19).

To investigate the functional connection between PGRMC1-S181-phosphorylation and increased proliferation, the proliferation of the phosphorylation deficient MCF7/PGRMC1-S181A cells upon treatment with progestins was investigated. In addition, we used the cell line MCF7/PGRMC1-S57A, which overexpresses the phosphorylation deficient PGRMC1 variant S57A, and the double-variant cell line MCF7/PGRMC1-S57A/S181A [34]. The phosphorylation site S57 was previously not found to be differentially phosphorylated upon NET treatment in MCF7/PGRMC1 cells [27] and therefore served as a control. In accordance with our previous findings, the proliferation of the control cell line MCF7/PGRMC1-S57A significantly increased after stimulation with PPPs (Figure 3D), similarly to MCF7/PGRMC1 cells, whereas the proliferation of MCF7/PGRMC1-S181A (Figure 3E) cells and MCF7/PGRMC1-S57A/S181A (Figure 3F) cells increased only after NET treatment. This result suggested that PGRMC1-S181-phosphorylation was important for the proliferative effect observed upon PGRMC1 overexpression and PPP treatment.

After demonstrating that PGRMC1-S181 phosphorylation accompanied the increase in proliferation observed after treatment with PPPs, we investigated whether this phosphorylation was crucial for the recruitment of PHBs to PGRMC1. Since the Co-IPs followed by mass spectrometry and Western blot indicated that PHB1 and PHB2 interacted with PGRMC1 upon treatment with NET, we next performed the Co-IP after treatment with both PPPs and N-PPPs and analyzed the precipitated proteins by Western blotting. Both PHB1 and PHB2 showed significantly higher abundance in PGRMC1 Co-IP pellets upon treatment with the PPPs NET, DYD and DSP and a clear trend for MPA compared to treatment with DMSO in MCF7/PGRMC1 (Figure 3G,H, Figures S4A,B and S16) and T47D/PGRMC1 cell lines (Figures S4C–F, S21 and S22). PHB1 and PHB2 association was especially strong for DYD-treated cells whereas PHB1 and PHB2 protein levels were similar in all cell lysates (Figure 3G, Figures S4A and S20). PHB1 or PHB2 levels in Co-IP precipitates after N-PPP treatments were not significantly different than control levels. Co-IPs with lysates of cells overexpressing phosphorylation-deficient PGRMC1-variants demonstrated that PGRMC1-S181 is crucial for the recruitment of PHBs to Co-IP pellets after treatment with PPPs. PHB1 (Figure 3I,J and Figure S17) and PHB2 (Figures S4G,H and S23) could be precipitated by PGRMC1 from lysates of the control cell line MCF7/PGRMC1-S57A but not from Co-IP pellets from MCF7/PGRMC1-S181A and MCF7/PGRMC1-S57A/S181A cells. Taken together, these results suggest that treatment with PPPs leads to PGRMC1-S181 phosphorylation and increased interaction of PHB1 and PHB2 with protein complexes containing PGRMC1.

### 3.4. PGRMC1-PHB1/PHB2 Association Diminishes PHB1/PHB2 Binding to ERα

**Figure 4:**
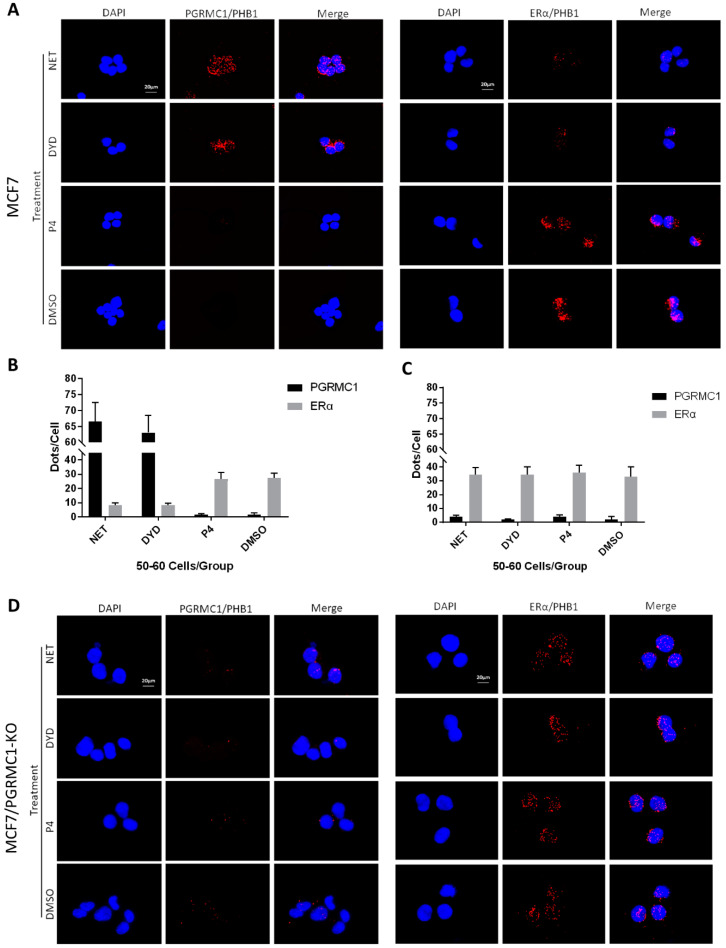
PGRMC1-PHBs interaction disturbs PHBs’ binding to ERα. (**A**) Proximity ligation assay (PLA) for PGRMC1- (or ERα-) interactions with PHB1 upon treatment with NET, DYD, P4 (10^−6^ M) and DMSO in MCF7 cells. Analysis of PLA for PGRMC1- (or ERα-) interactions with PHB1 in (**B**) MCF7 and (**C**) MCF7/PGRMC1-KO cells upon treatment with progestins and DMSO. Dots per cell were counted for 50-60 cells in each sample. Cell number and PLA signals were quantified using imageJ software. (**D**) PLA for PGRMC1- (or ERα-) interactions with PHB1 upon treatment with progestins and DMSO in MCF7/PGRMC1-KO cells. Each red spot represents a single interaction. Nuclear stain: DAPI. Magnification 40×.

Since PHB1 and PHB2 were reported to regulate ERα signaling, which is a central oncogenic pathway in luminal breast cancer, we focused on the implication of PGRMC1 in the ERα signaling network and its possible involvement in breast cancer promotion. According to literature, PHB2 directly interacts with ERα and represses its transcriptional activity [39]. Therefore, we investigated the associations between endogenously expressed PGRMC1 and PHBs or ERα and PHBs, respectively, by PLA in parental MCF7 and T47D cells-independent of overexpression and immunoprecipitation. For this experiment, NET and DYD were used representatively for the PPP group while P4 and DMSO served as controls. Upon treatment with NET and DYD, a significantly higher number of PLA interactions between PGRMC1 and PHB1 (Figure 4A,B) or PHB2 (Figure S5A,B) could be observed in both cell lines compared to treatment with P4 and DMSO (T47D in Figure S7A–D).

Regarding the interaction between ERα and PHB1 or PHB2, we obtained the inverse picture: treatment with NET or DYD led to significantly less interactions than the treatment with P4 or DMSO (Figure 4A,B and Figure S5A,B, T47D in Figure S7A–D), while the protein expression levels of all tested proteins remained unchanged (Figure S10).

To test whether the decrease of associations between PHBs and ERα after treatment with NET and DYD is conveyed by PGRMC1, we established PGRMC1 deficient MCF7 cells (MCF7/PGRMC1-KO) using the CRISPR/Cas9 approach. As control, we used cells that were treated with the respective control plasmid (expressing an unspecific gRNA) and analogously expanded from single cell clones (MCF7/PGRMC1-KO/Control). In MCF7/PGRMC1-KO cells that have been selected by PCR screening of single cell clones, PGRMC1 expression was below detection level as tested by Western blotting using two different antibodies (Figures S6, S24 and S25). The PLA in MCF7/PGRMC1-KO cells demonstrated that in the absence of PGRMC1, the interaction between ERα and PHBs did not change with progestin treatment (Figure 4C,D and Figure S5C,D), while MCF7/PGRMC1-KO/Control cells behaved similarly to parental MCF7 cells (Figure S8A–D), implying a dependence upon PGRMC1 in the progestin-dependent release of ERα from PHBs. For isotype controls of the PLA reaction, see Figure S9A,B.

### 3.5. ERα Is Activated upon Progestin-Treatment in a PGRMC1-Dependent Manner

**Figure 5:**
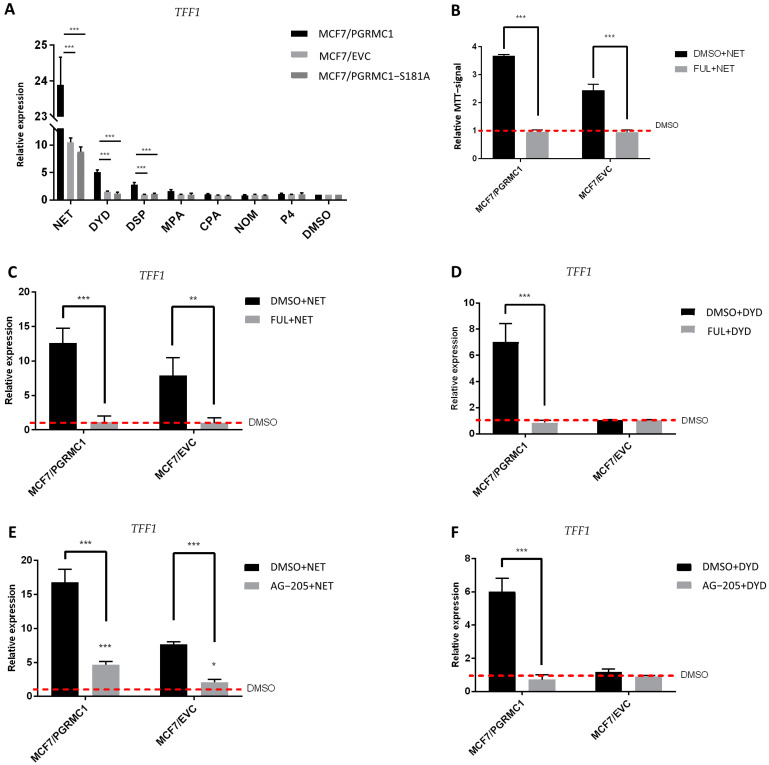
ERα is activated upon progestin-treatment in a PGRMC1-dependent manner. (**A**) qRT-PCR analysis of *TFF1* mRNA expression in MCF7/PGRMC1, MCF7/EVC and MCF7/PGRMC1-S181A cells upon treatment with progestins (10^−6^ M) or DMSO (0.01%) for 24 h. (**B**) Relative MTT signal as surrogate for cell number of MCF7/PGRMC1 and MCF7/EVC cells treated with fulvestrant (10^−7^ M) and NET (10^−6^ M) or DMSO (0.01%). Values were normalized to DMSO treated cells. qRT-PCR analysis of *TFF1* mRNA expression in MCF7/PGRMC1 and MCF7/EVC cells upon treatment with (**C**) fulvestrant (10^−7^ M) and NET (10^−6^ M), (**D**) fulvestrant and DYD (10^−6^ M), (**E**) AG-205 (25 × 10^−6^ M) and NET, (**F**) AG-205 and DYD, or DMSO, respectively. Signal intensity was normalized to respective DMSO control. Statistical analysis was performed by two-way ANOVA and Bonferroni post-hoc test. *: *p* < 0.05, **: *p* < 0.01, ***: *p* < 0.001.

After demonstrating that ERα is released from PHBs upon binding of PGRMC1 to the latter after stimulation with PPPs, we speculated that progestin-dependent PGRMC1 interaction with PHB1 and PHB2 might result in elevated ERα activation and subsequent increase in ERα target gene expression. To examine this hypothesis, we analyzed the mRNA expression level of trefoil factor 1 (*TFF1*) as a reporter gene for ERα activation upon treatment with both PPPs and N-PPPs in MCF7/PGRMC1 as well as in T47D/PGRMC1 cells and their respective EVC cells. Additionally, we stimulated MCF7/PGRMC1-S181A cells, as the S181-phosphorylation site is critical for the interactions between PGRMC1 and PHBs. We observed increased expression of *TFF1* upon treatment with PPPs in both MCF7/PGRMC1 (Figure 5A) and T47D/PGRMC1 (Figure S11) cells compared to DMSO control, whereas treatment with N-PPPs did not result in any significant differences. Additionally, expression of *TFF1* in MCF7/PGRMC1-S181 cells did not vary upon treatment with any progestin relative to MCF7/EVC control cells (Figure 5A), emphasizing the importance of PGRMC1-S181 phosphorylation in progestin-dependent ERα activation.

Further, in order to verify that the proliferative effect observed after treatment with PPPs correlated with *TFF1* expression and ERα activation, we pre-treated the same cell lines with the selective ERα-degrader fulvestrant [35] before the stimulation with NET or DYD. As expected, degradation of ERα before progestin treatment (for confirmation of ERα-degradation, see Figures S12, S26 and S27) resulted in diminished *TFF1* expression (Figure 5C,D). In addition, the proliferative effect of NET was completely abolished after pre-treatment with fulvestrant, validating that the progestin-dependent increased breast cancer cell viability is conveyed through the ERα signaling pathway (Figure 5B). To further elucidate the role of PGRMC1 in progestin-dependent ERα activation, we pre-treated MCF7/PGRMC1 and MCF7/EVC cells with the putative PGRMC1 inhibitor AG-205 [45,46]. AG-205 antagonizes some PGRMC1 functions but also has PGRMC1-independent effects [47]. Treatment with AG-205 before the stimulation with NET or DYD representatively for PPPs completely abolished the increase of *TFF1* expression in both cell lines for DYD and significantly diminished the same for NET, indicating an essential role of PGRMC1 in progestin-dependent ERα activation (Figure 5E,F).

## 4. Discussion

Various studies have demonstrated that elevated PGRMC1 expression promotes a more aggressive phenotype of breast cancer and participates in its carcinogenesis [48,49]. High expression of PGRMC1 correlates with poor outcome, which has been reported for breast-, lung-, ovarian- and kidney cancer [48,50,51,52]. In previous studies, we demonstrated that PGRMC1 is partially required for progestin signaling in MCF7 cells and therefore suggested a potential role of PGRMC1 in the increased breast cancer risk upon progestin-based HT [27,28,29].

Our present study focuses on a progestin-dependent crosstalk between PGRMC1 and ERα signaling in ERα/PR positive breast cancer cells. Our findings suggest the function of PGRMC1 as an important amplifier of ERα-dependent transcription upon treatment with the PPPs NET, DYD, DSP, and MPA, resulting in oncogenic signaling and tumor progression in ERα positive breast cancer cells. These results are in accordance with a study by Ruan et al., who also detected proliferation-enhancing effects of NET, DYD, DSP, and MPA, whereas no effect could be observed for NOM or P4 [31]. They are further supported by a recently published xenograft study by Zhao et al., who found higher tumor volumes of PGRMC1 overexpressing MCF7 and T47D cells in NET treated mice compared to tumor volumes of the respective EVC cells [30].

To identify responsible factors that are involved in oncogenic signaling of HR positive breast cancer cells, we performed Co-IP with HA-PGRMC1 followed by mass spectrometry and identified PHB1 and PHB2 as possible PGRMC1 interaction partners upon treatment with NET. We do not demonstrate direct physical interactions, however at the very least PGRMC1 and PHBs are present in the same Co-IP pellets and are sufficiently proximal to permit a positive PLA signal. As reviewed by Cahill et al., PGRMC1 phosphorylation could play a crucial role not only in terms of its function but also for its protein-protein interactions and subcellular localization [44]. In a previous study, we investigated PGRMC1 phosphorylation in MCF7 breast cancer cells after treatment with NET and identified S181 to be phosphorylated [27].

In the current investigation, we further demonstrate that PGRMC1-S181-phosphorylation is promoted by PPPs and correlates with increased proliferation of treated cells. We observed increased phosphorylation at S181 for both exogenously and endogenously expressed PGRMC1 after treatment with PPPs. Hence, ablation of the S181 phosphorylation site by single amino acid substitution to alanine or in combination with S57A significantly diminished proliferation of MCF7/PGRMC1-S181A and MCF7/PGRMC1-S57A/S181A cells. In addition, we demonstrated that PGRMC1-S181 phosphorylation is essential for association of PGRMC1 with PHBs after treatment with all PPPs and that the PGRMC1-PHB association is abolished in PGRMC1-S181 phosphorylation-deficient cells. According to these findings, we assume that phosphorylation of PGRMC1 at S181 is crucial for its downstream signaling and the resulting increase in cell proliferation upon progestin treatment. Future studies should address the role, if any, of the adjacent Y180 residue in PHB interactions. Mutation of Y180 in MIA PaCa-2 pancreatic cancer cells reduced signaling by the PI3K/Akt pathway, accompanied by large metabolic and epigenetic changes [43,53].

Both PHBs are reported to exert various functions depending on their localization in the cell and can act independently as well as in a heterodimeric complex [32]. In addition to being a scaffold for mitochondrial proteins in the inner mitochondrial membrane [32], both PHBs have been described as transcription factor modulators which interact with various transcription factors in the nucleus, particularly with ERα [32,39,40]. For luminal breast cancer, PHB2 has been discussed as a potential tumor suppressor since its overexpression significantly diminished ERα signaling, whereas its downregulation elevated the latter [40].

**Figure 6:**
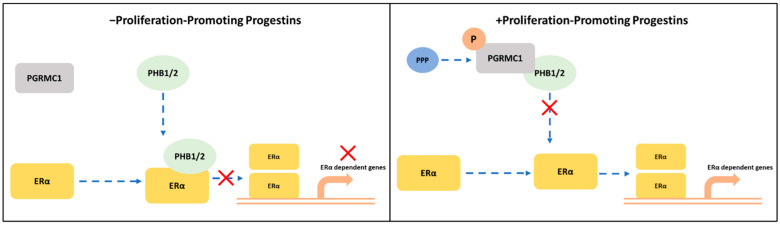
Potential crosstalk between PGRMC1 and PHB1/2 in ERα-signaling cascades. S181-phosphorylation on PGRMC1 mediates interaction with PHB1/2 upon treatment with proliferation-promoting progestins (PPPs). In absence of PPPs: PHB1/2 act as ERα co-regulators to inhibit the transcription of ERα-dependent genes. In presence of PPPs: S181-phosphorylated PGRMC1 interacts with PHB1/2, possibly inhibiting their function as transcription factor regulators and enabling the transcription of ERα-dependent genes.

In the present study, we demonstrated that PGRMC1 may represent a regulating factor in the PHBs-ERα-interplay. Stimulation with PPPs increased the association between PGRMC1 and PHBs, which reduced the interactions between PHBs and ERα. A potential mechanism may be that this results in a reduced capability of PHBs inhibit ERα transcriptional activity in the promoter regions of ER-target genes (Figure 6). Indeed, the PLA between the different proteins using NET and DYD representatively for the group of PPPs and P4 and DMSO as controls revealed significantly reduced interactions between ERα and PHBs and significantly elevated ERα activation (measured as *TFF1* transcription level) upon treatment with NET and DYD. This finding points towards an indirect stimulatory effect on ERα by PGRMC1 via neutralization of the inhibitory effect of PHBs. A similar role has been reported for the brefeldin A-inhibited guanine nucleotide-exchange protein 3 (BIG3) which binds PHB2 to prevent its translocation into the nucleus, and thereby acts as an ERα coactivator [54,55,56].

Consistently with this model, pharmacological inhibition of each of PGRMC1 by AG-205 or ERα by fulvestrant annulled the stimulatory effect of DYD and significantly diminished the same for NET, substantiating the assumption that both PGRMC1 and ERα essentially contribute to the propagation of progestin signals in breast cancer. Interestingly, in a recent publication, Teakel and coauthors identified PHBs as PGRMC1 interaction partners in the pancreatic cancer cell line MIA PaCa-2 independently of progestin treatment [33], pointing towards an implication of PGRMC1 in PHB1/PHB2- function that is not limited to breast cancer or progestin stimulation, which deserves further investigation. This is especially interesting considering that PHBs regulate additional transcription factors to ERα, e.g., E2F1, p53 [57,58], and implicates new ways that PGRMC1 might modulate the context of oncogenic signaling and apoptosis in other tumor settings.

Concerning the inhibitory function of AG-205 on PGRMC1, it is important to mention that not all the effects observed upon AG-205 treatment in the literature appear to be PGRMC1-specific. AG-205 has been repeatedly used by several research groups as mutual PGRMC1 inhibitor to confirm the role of PGRMC1 in membrane trafficking and epithelial growth factor receptor (EGFR) activation, activation of glucagon-like peptide 1 receptor and fatty acid 2-hydrolase [50,59,60,61]. However, as recently demonstrated by Wang-Eckhardt et al., formation of large vesicular structures in response to AG-205 treatment occurred independently of PGRMC1 expression [47]. Furthermore, in endometrial cells, AG-205 treatment led to increased expression of genes involved in cholesterogenesis and steroidogenesis, both independently of PGRMC1 expression [62]. These two findings emphasize that caution is advised when using AG-205 as a mutual PGRMC1 inhibitor. Although the binding of AG-205 to PGRMC1 has been demonstrated [50], its exact mechanism of action and possible activity on other targets remains uncharacterized.

In our experiments, the strongest proliferative effect and the highest increase of *TFF1* expression was measured after NET stimulation, with DYD being the second most potent progestin. However, as to PGRMC1-S181 phosphorylation and the interaction between PGRMC1 and PHBs, we found the highest level of PGRMC1-phosphorylation and the strongest increase of PHB-interactions for DYD.

Concerning this issue, it is important to mention that in T47D cells, NET was previously shown to be bioconverted into the ERα-agonists 3α,5α-norethisterone and 5α-norethisterone [63]. Hence, besides activation of PGRMC1 and associated downstream targets, metabolites of NET might also directly bind to ERα, facilitating ligand-dependent ERα signaling. This is consistent with the observation that inhibition of PGRMC1 using AG-205 resulted in a completely abolished increase of *TFF1* expression when treated with DYD, whereas treatment with NET was accompanied by a significantly decreased but still measurable ERα activation, perhaps through direct ERα binding by NET metabolites.

In an earlier publication dealing with increased breast cancer risk for women receiving combined hormone therapy, we used MPA in combination with E2 and reported that MPA sensitized PGRMC1-overexpressing MCF7 cells to E2 at low concentration [64]. Further, in in vivo studies a sequential combined treatment of E2 and NET significantly increased tumor growth of MCF7/PGRMC1 cells, compared to E2-only treatment [29]. In the present study, we treated the cells with the progestins alone in hormone free medium. Nevertheless, as previously described, we measured increased production of estradiol in MCF7/PGRMC1 cells [24], indicating that these cells might endogenously activate ERα even in the absence of exogenous E2. Therefore, the exact mechanism of ERα activation and recruitment to the *TFF1* promotor needs further investigation.

Our data rather describes the impact of PGRMC1 on PHBs’ function as transcription factor modulators and contributes to revealing the PGRMC1 regulatory network with special focus on processes driving breast cancer progression. It is of notable interest that nuclear steroid receptor transcription factors, of which ERα was the first in chordates, evolved in bilaterian animals [65]. Bilaterians concomitantly gained the cognate of the T178/Y180/S181 module in the eumetazoan PGRMC C-terminus [66], suggesting that the processes we describe here may reflect ancient bilaterian biology that is perturbed in cancer. Future studies should further elucidate the mechanism of elevated ERα activation mediated by PGRMC1 to shed light on the regulation of this oncogenic signaling pathway. The interaction partners detected in the present study will be an important starting point to further investigate the PGRMC1 signaling cascade in HR positive breast cancer.

Since activated PGRMC1 may potentiate the oncogenic signaling of ERα and thereby promote breast cancer progression, it may serve as a therapeutic target. In this context, Kabe et al. recently identified glycyrrhizin, a major component in licorice extract with anti-inflammatory and anti-viral effects [67], as a substance that directly binds PGRMC1 and inhibits some of its functions [68]. In a human colon cancer cell line, glycyrrhizin inhibited the interaction of PGRMC1 with EGFR, suppressing EGFR signaling and increasing chemosensitivity towards erlotinib and cisplatin [68]. Future studies should investigate the effect of glycyrrhizin on PGRMC1 in the context of breast cancer. Given the potential mechanism presented in the current study, the possible pharmacologic inhibition of PGRMC1 in combination with antihormonal treatment could be of high interest.

## 5. Conclusions

In the present study, we identified PGRMC1 as a factor that inhibits PHBs’ action as ERα co-regulators in the presence of certain progestins in our luminal breast cancer cell model. PGRMC1 is thereby involved in a key oncogenic signaling pathway in breast cancer. Our data underline the contribution of PGRMC1 to especially hormone receptor positive breast cancer pathogenesis and demonstrate the urgent need for further studies.

## Data Availability

Relevant data supporting the findings of this study are available within the article and Supplementary Materials and are available from the authors upon reasonable request. The mass spectrometry proteomics data have been deposited to the ProteomeXchange Consortium via the PRIDE [37] partner repository with the dataset identifier PXD028537.

## Supplementary Materials

The following are available online at https://www.mdpi.com/article/10.3390/cancers13225635/s1, Figure S1: Cell viability of breast cancer cells upon progestin treatment, Figure S2: Relative amount of precipitated PGRMC1, Figure S3: PGRMC1 is phosphorylated at S181 upon treatment with proliferation-promoting progestins, Figure S4: PHB1 and PHB2 are precipitated by PGRMC1 after treatment with PPPs, Figure S5: PLA for PGRMC1 and PHB2 or ERα and PHB2 in MCF7 cells, Figure S6: PGRMC1 protein level in parental MCF7 and PGRMC1-knockout cells, Figure S7: PLA for PGRMC1 and PHB1/PHB2 or ERα and PHB1/PHB2 in T47D cells, Figure S8: PLA for PGRMC1 and PHB1/PHB2 or ERα and PHB1/PHB2 in MCF7/PGRMC1-KO/Control cells, Figure S9: Negative control PLA using isotype antibodies, Figure S10: Immunofluorescence staining of MCF7 cells for PGRMC1, PHB1, ERα and PHB2, Figure S11: qRT-PCR analysis of *TFF1* mRNA expression in T47D/PGRMC1 and T47D cells, Figure S13. Left panel: analysis of immunopurified (HA-based) precipitate from PGRMC1 overexpressing cell lines MCF7/PGRMC1 and MCF7/PGRMC1-GFP cells; detection of PHB1 and PGRMC1. Right panel: Protein level in whole cell, Figure S14. Left panel: analysis of immunopurified (HA-based) precipitate from PGRMC1 overexpressing cell lines MCF7/PGRMC1 and MCF7/PGRMC1-GFP cells; detection of PHB2 and PGRMC1. Right panel: Protein level in whole cell, Figure S15. Analysis of PGRMC1-S181-phosphorylation after progestin treatment in whole cell lysates of MCF7/PGRMC1 cells. Upper panel: detection of S181-phosphorylated PGRMC1; Lower panel: detection of PGRMC1 protein level, Figure S16. Analysis of immunopurified (HA-based) precipitate from MCF7/PGRMC1 cells after progestin treatment (upper panel) and protein level in whole cell lysates of the same samples (lower panel). Detection of PHB1, Figure S17. Analysis of immunopurified (HA-based) precipitate from MCF7 cells overexpressing PGRMC1 or phosphorylation-deficient PGRMC1 variants after treatment with DYD. Detection of PHB1, Figure S18. Analysis of PGRMC1-S181-phosphorylation after progestin treatment in whole cell lysates of MCF7/EVC cells. Upper panel: detection of S181-phosphorylated PGRMC1; Lower pan-el: detection of PGRMC1 protein level, Figure S19. Analysis of PGRMC1-S181-phosphorylation after progestin treatment in whole cell lysates of MCF7/PGRMC1-S181A cells. Upper panel: detection of S181-phosphorylated PGRMC1; Lower panel: detection of PGRMC1 protein level, Figure S20. Analysis of immunopurified (HA-based) precipitate from MCF7/PGRMC1 cells after progestin treatment (upper panel) and protein level in whole cell lysates of the same samples (lower panel). Detection of PHB2, Figure S21. Analysis of immunopurified (HA-based) precipitate from T47D/PGRMC1 cells after progestin treatment (upper panel) and protein level in whole cell lysates of the same samples (lower panel). Detection of PHB1, Figure S22. Analysis of immunopurified (HA-based) precipitate from T47D/PGRMC1 cells after progestin treatment (upper panel) and protein level in whole cell lysates of the same samples (lower panel). Detection of PHB2, Figure S23. Analysis of immunopurified (HA-based) precipitate from MCF7 cells overexpressing PGRMC1 or phosphorylation-deficient PGRMC1 variants after treatment with DYD. Detection of PHB2, Figure S24. Analysis of PGRMC1-expression in whole cell lysates from MCF7, MCF7/PGRMC1-KO/Control and MCF7/PGRMC1-KO cells. Detection of PGRMC1 with the N-terminal antibody (Cell Signaling), Figure S25. Analysis of PGRMC1-expression in whole cell lysates from MCF7, MCF7/PGRMC1-KO/Control and MCF7/PGRMC1-KO cells. Detection of PGRMC1 with the C-terminal antibody (Abcam), Figure S26. Analysis of ERα- and PGRMC1-protein level in MCF7/PGRMC1 and MCF7/EVC cells after treatment with fulvestrant and NET, Figure S27. Analysis of ERα- and PGRMC1-protein level in MCF7/PGRMC1 and MCF7/EVC cells after treatment with fulvestrant and DYD, Table S1: List of proteins identified in the precipitate by mass spectrometry after HA-based enrichment of PGRMC1 from whole cell lysates of MCF7/PGRMC1 and MCF7/PGRMC1-GFP cells treated with NET (10^−6^ M) or DMSO.

## Appendix A

Table S1: List of proteins identified in the precipitate by mass spectrometry after HA-based enrichment of PGRMC1 from whole cell lysates of MCF7/PGRMC1 and MCF7/PGRMC1-GFP cells treated with NET (10^−6^ M) or DMSO. In addition to the data on the statistical analysis, the tables contain mean values of LFQ-intensity values for the different groups and ratios of selected mean values as indicated. Additionally relevant protein identification and quantification data from MaxQuant is shown. For details please refer to Tyanova et al., 2016 [69]. List of 701 co-precipitated proteins (Table S1 701 Proteins) identified by mass spectrometry from HA-immunopurified proteins of MCF7/PGRMC1 and MCF7/PGRMC1-GFP cells treated with NET (10^−6^ M) or DMSO. GFP DMSO: immunopurified samples of DMSO treated MCF7/PGRMC1-GFP cells. GFP NET: immunopurified samples of NET treated MCF7/PGRMC1-GFP cells. HA DMSO: immunopurified samples of DMSO treated MCF7/PGRMC1 cells. HA NET: immunopurified samples of DMSO treated MCF7/PGRMC1 cells. Potential PGRMC1 interacting proteins were selected by a ratio HA/GFP > 2 and additionally a corrected *p*-value < 0.01 for the variable “TAG” in the 2 way-ANOVA. For the 253 remaining proteins (Table S1 253 Proteins) additionally Welch-tests were performed (significance analysis of microarrays, S0 = 0.1, 1% false discovery rate, Tusher et al., 2001) [70] comparison of NET and DMSO treated HA-PGRMC1 samples.
