# Supplementary material for: PGRMC1 Promotes Progestin-Dependent Proliferation of Breast Cancer Cells by Binding Prohibitins Resulting in Activation of ERα Signaling
Source: Cancers (Basel). 2021 Nov 11;13(22):5635. doi: 10.3390/cancers13225635 (PMC8615993; doi:10.3390/cancers13225635)
Supplement: Supplementary file 1 [file cancers-13-05635-s001.zip › cancers-1439041-supplement Figures.pdf]

# Supplementary Materials: PGRMC1 promotes progestin-dependent proliferation of breast cancer cells by binding prohibitins resulting in activation of ERα signaling

**Yingxue Bai, Marina Ludescher, Gereon Poschmann, Kai Stühler, Martine Wyrich, Julia Oles, André Franken, Mahdi Rivandi, Anna Abramova, Florian Reinhardt, Eugen Ruckhäberle, Dieter Niederacher, Tanja Fehm, Michael A. Cahill, Nadia Stamm and Hans Neubauer**

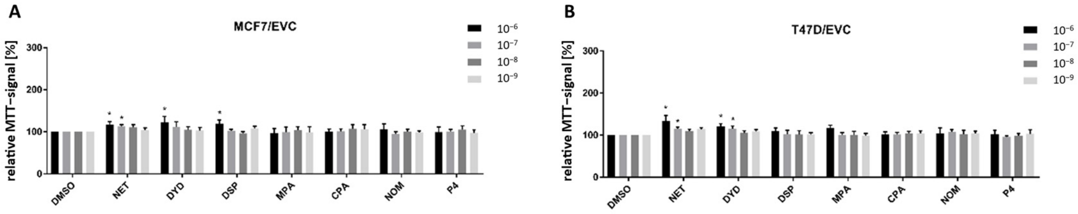


**Figure S1.** Cell viability of breast cancer cells upon progestin treatment. Cell viability of (**A**) MCF7/EVC and (**B**) T47D/EVC cells. Cells were grown in different concentrations of progestins ($10^{-6}$–$10^{-9}$ M) for 72 h. Values were normalized to DMSO treated cells. Statistical analysis was performed with two-way ANOVA and Bonferroni post–hoc test. *: $p < 0.05$.

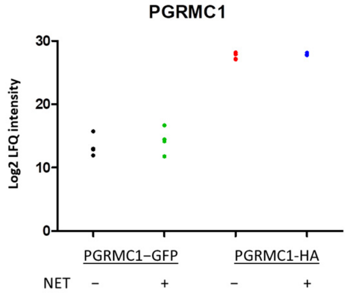


**Figure S2.** Relative amount of precipitated PGRMC1. Analysis of immunopurified (HA–based) samples of MCF7/PGRMC1–GFP cells (PGRMC1–GFP) and MCF7/PGRMC1 cells (PGRMC1–HA) treated with DMSO or NET ($10^{-6}$ M) for co–precipitated proteins. Mass spectrometry results for precipitated PGRMC1, log2 normalized intensity.

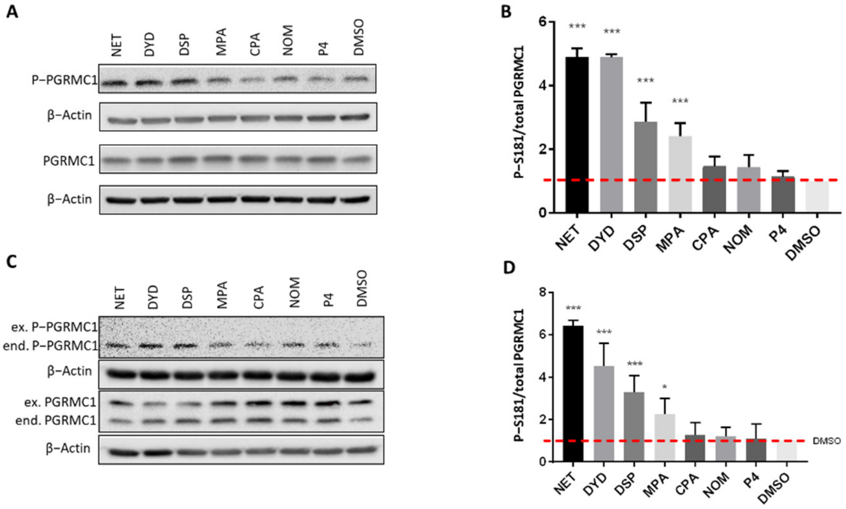

**Figure S3.** PGRMC1 is phosphorylated at S181 upon treatment with proliferation−promoting progestins. (**A**−**D**) Analysis of PGRMC1−S181−phosphorylation and PGRMC1 protein levels by Western blot of whole cell lysates of (**A**) MCF7/EVC and (**C**) MCF7/PGRMC1−S181A cells after treatment with progestins ($10^{-6}$ M) or DMSO. S181−phosphorylation occurs only on the endogenous PGRMC1 (lower band, ≈25kDa). Densitometric analysis of Western blot results for S181−phosphorylation intensity of endogenous PGRMC1 in (**B**) MCF7/EVC and (**D**) MCF7/PGRMC1−S181A. Intensity was normalized to corresponding DMSO control. Statistical analysis was performed with one−way ANOVA and Bonferroni post−hoc test. **: $p < 0.01$, ***: $p < 0.001$.

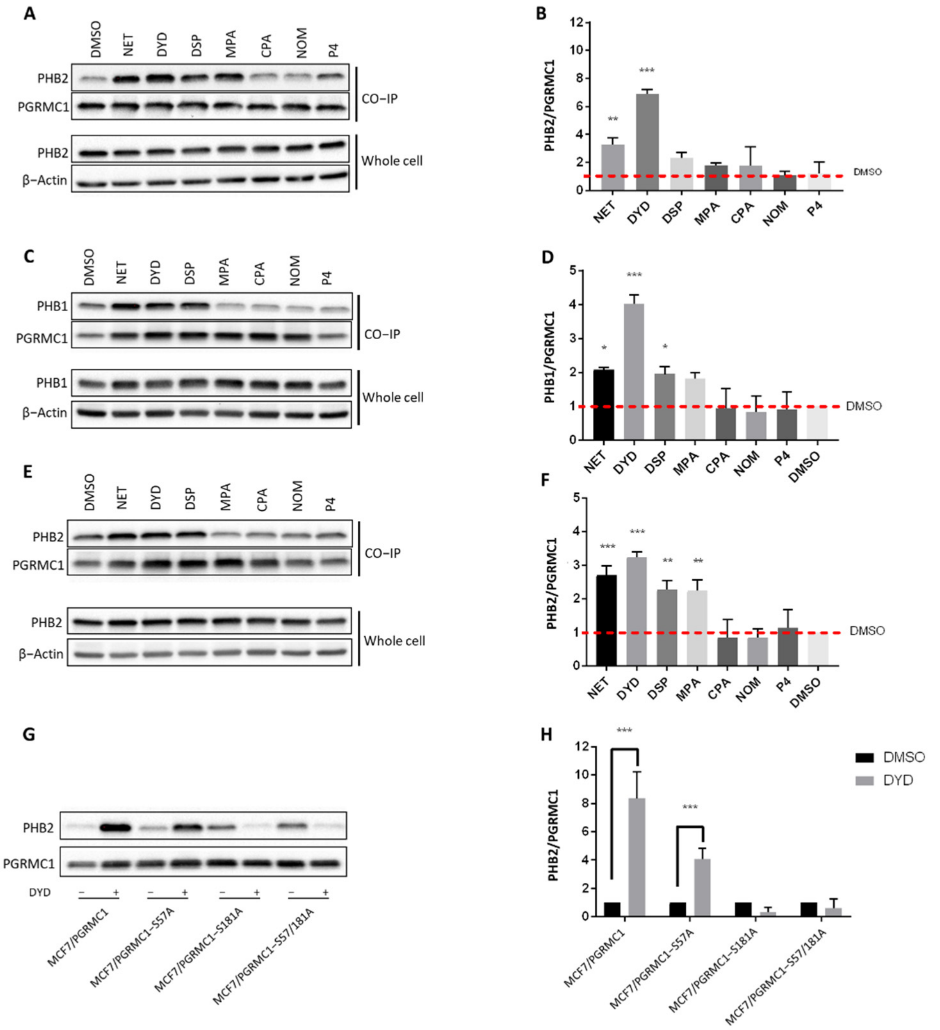


**Figure S4.** PHB1 and PHB2 are precipitated by PGRMC1 after treatment with PPPs. (**A**) Western blot analysis of immunopurified HA−tagged PGRMC1 and co−precipitated PHB2 from MCF7/PGRMC1 cells treated with progestins ($10^{-6}$ M) and DMSO (upper panel) and PHB2 protein levels in whole cell lysates in the same cells (lower panel). (**B**) Densitometric analysis of co−precipitated PHB2. (**C**−**F**) Analysis of PGRMC1−PHB1/2 interactions upon treatment with progestins (all $10^{-6}$ M) or DMSO in T47D/PGRMC1 cells. Western blot analysis of immunopurified HA−tagged PGRMC1 and co−precipitated (**C**) PHB1 and (**E**) PHB2 (each upper panel) and of the PHB1 and PHB2 protein levels in the respective whole cell lysates (each lower panel). Densitometric analysis for (**D**) PHB1 and (**F**) PHB2. (**G**) Western blot analysis of immunopurified HA−tagged PGRMC1−variants and co−precipitated PHB2 after treatment with DYD ($10^{-6}$ M) or DMSO. (**H**) Densitometric analysis of co−precipitated PHB2. (**B**,**D**,**F**,**H**) Signal intensity was normalized to corresponding DMSO−control and signal intensity of each precipitated PGRMC1−variant. Statistical analysis was performed by one−way ANOVA (**A**−**F**) or two−way ANOVA (**H**) and Bonferroni post−hoc tests. *: $p < 0.05$, **: $p < 0.01$, ***: $p < 0.001$.

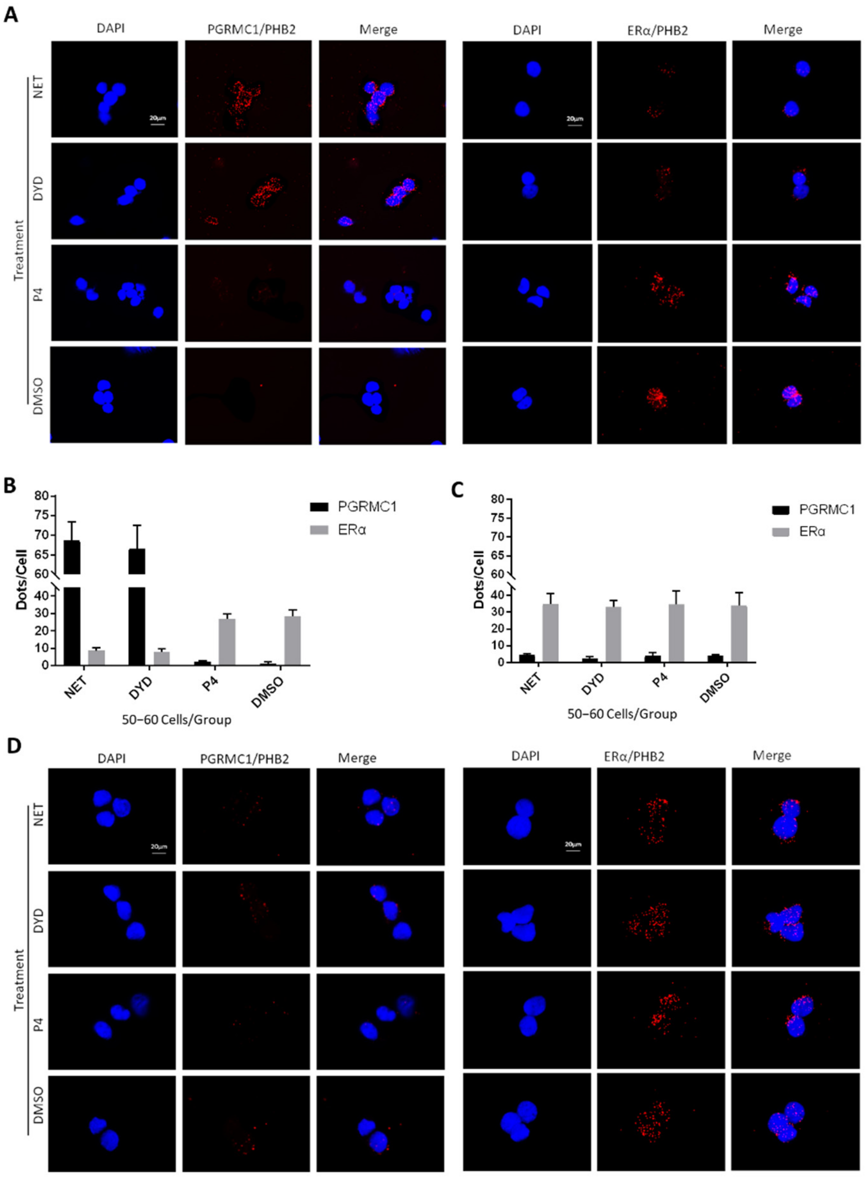


**Figure S5.** PLA for PGRMC1 and PHB2 or ER$\alpha$ and PHB2 in MCF7 cells. (**A**) PLA for PGRMC1− (or ER$\alpha$−) interactions with PHB2 upon treatment with NET, DYD, P4 ($10^{-6}$ M) and DMSO in MCF7 cells. Analysis of PLA for PGRMC1− (or ER$\alpha$−) interactions with PHB2 in (**B**) MCF7 cells and (**C**) MCF7/PGRMC1−KO cells upon treatment with progestins or DMSO. Dots per cell were counted for 50–60 cells in each sample. Cell number and PLA signals were quantified using imageJ software. (**D**) Proximity ligation assay (PLA) for PGRMC1− (or ER$\alpha$−) interactions with PHB2 upon treatment with progestins or DMSO in MCF7/PGRMC1−KO cells. Each red spot represents a single interaction. Nuclear stain: DAPI. Magnification 40×.

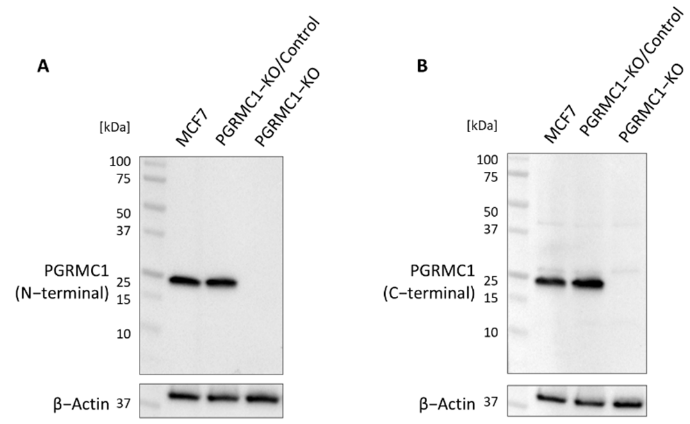


**Figure S6.** PGRMC1 protein level in parental MCF7 and PGRMC1−knockout cells. Western blot for PGRMC1 protein level in whole cell lysates of MCF7, MCF7/PGRMC1−KO/Control and MCF7/PGRMC1−KO cells. PGRMC1 protein was detected using (**A**) an N−terminal antibody (Cell Signaling) and (**B**) a C−terminal antibody (Abcam). Cells were cultured in complete medium.

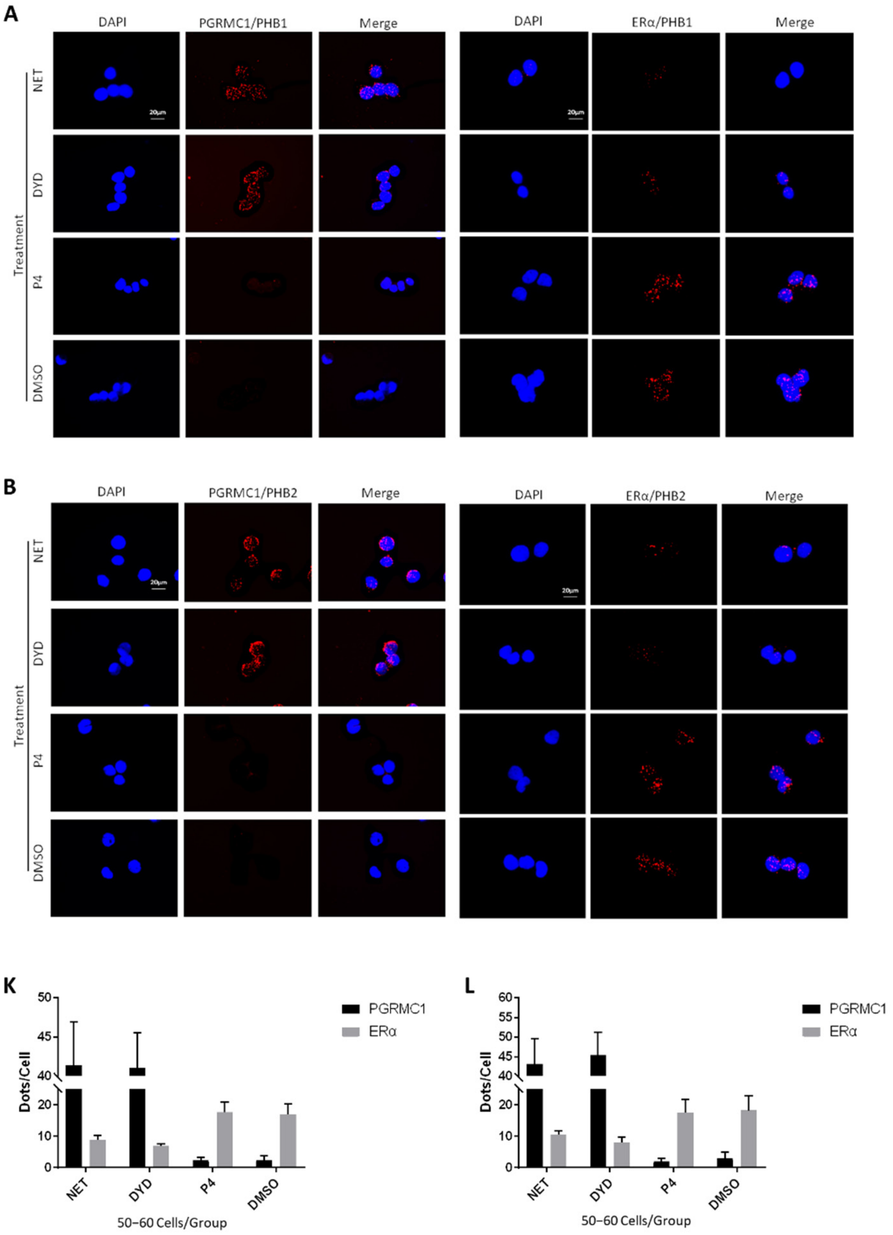


**Figure S7.** PLA for PGRMC1 and PHB1/PHB2 or ER*α* and PHB1/PHB2 in T47D cells. PLA for PGRMC1− (or ER*α*−) interactions with (**A**) PHB1 and (**B**) PHB2 upon treatment with NET, DYD, P4 ($10^{-6}$ M) or DMSO (0.01%) in T47D cells. Each red spot represents a single interaction. Nuclear stain: DAPI. Magnification 40×. Analysis of PLA for PGRMC1− (or ER*α*−) interactions with (**C**) PHB1 and (**D**) PHB2 in T47D cells upon treatment with progestins. Dots per cell were counted for 50–60 cells in each sample. Cell number and PLA signals were quantified using imageJ software.

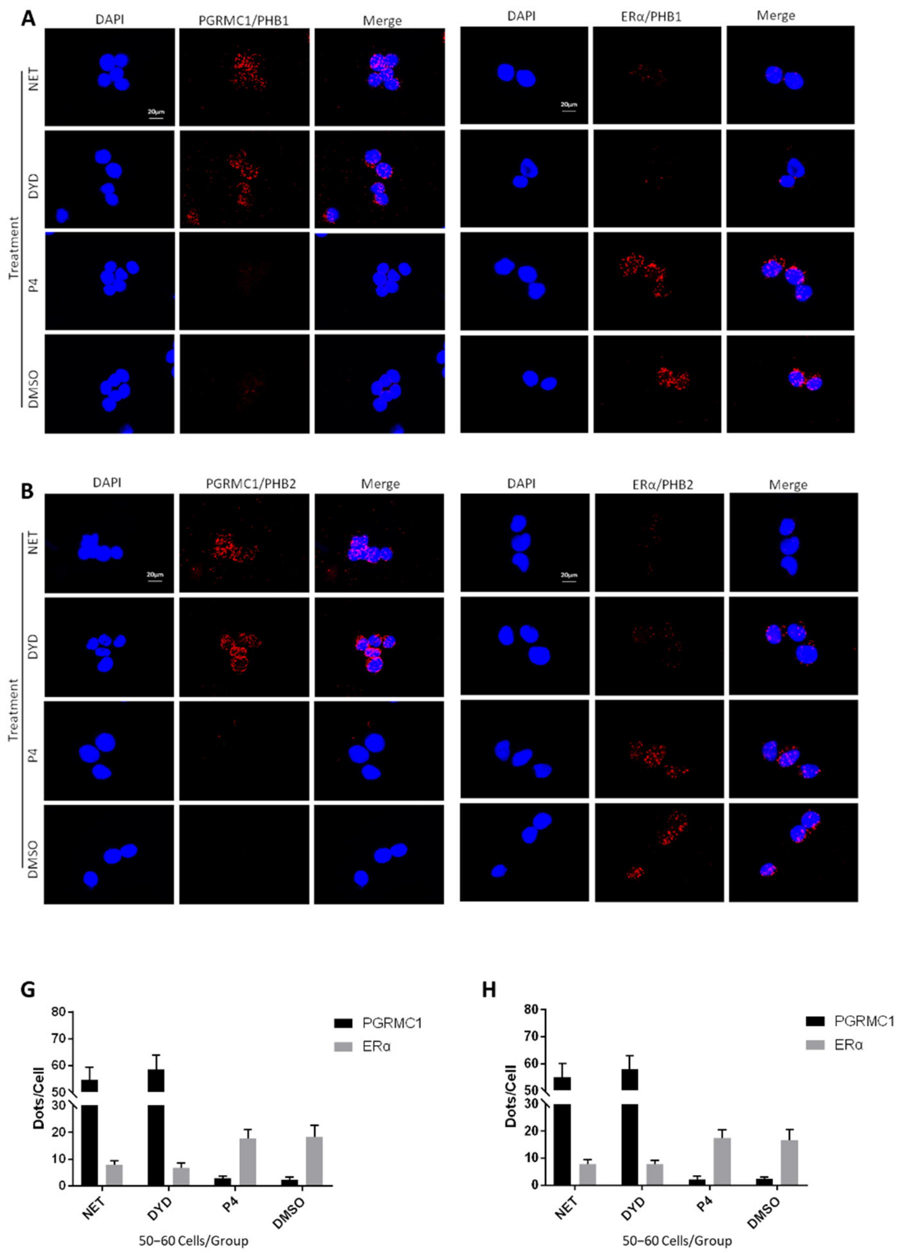


**Figure S8.** PLA for PGRMC1 and PHB1/PHB2 or ER$\alpha$ and PHB1/PHB2 in MCF7/PGRMC1−KO/Control cells. PLA for PGRMC1− (or ER$\alpha$−) interactions with (**A**) PHB1 and (**B**) PHB2 upon treatment with NET, DYD, P4 ($10^{-6}$ M) or DMSO (0.01%) in MCF7/PGRMC1−KO/Control cells. Each red spot represents a single interaction. Nuclear stain: DAPI. Magnification 40×. Analysis of PLA for PGRMC1− (or ER$\alpha$−) interactions with (**C**) PHB1 and (**D**) PHB2 in MCF7/PGRMC1−KO/Control cells upon treatment with progestins. Dots per cell were counted for 50–60 cells in each sample. Cell number and PLA signals were quantified using imageJ software.

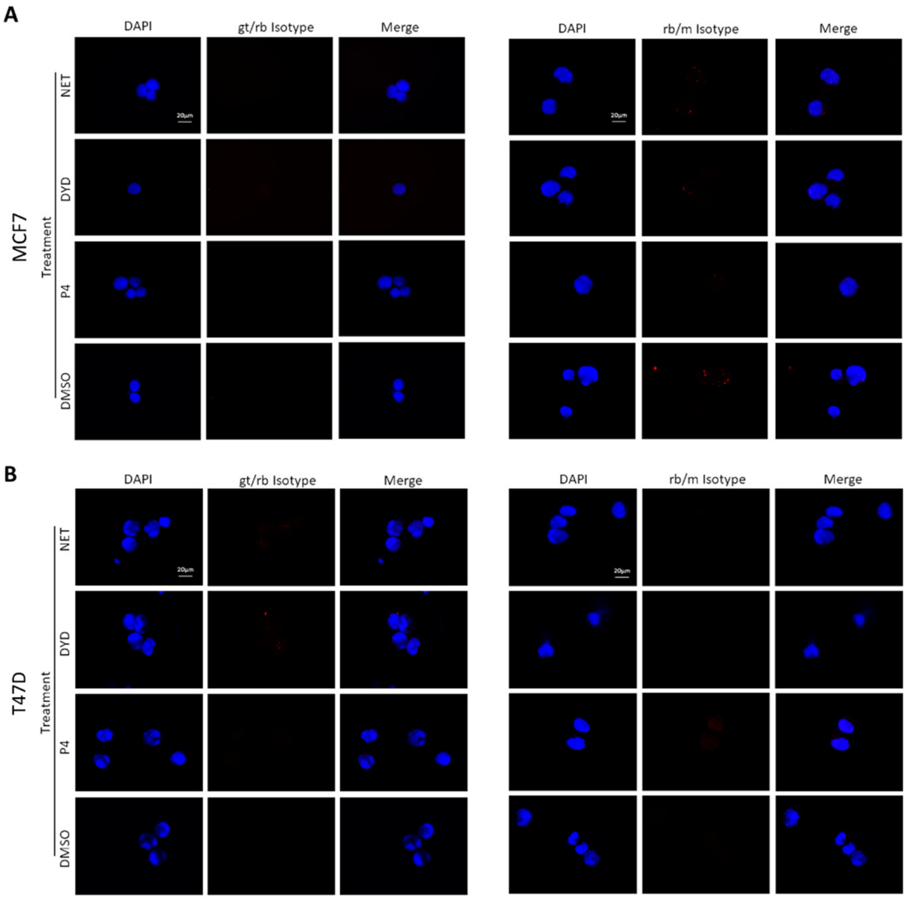


**Figure S9.** Negative control PLA using isotype antibodies. Negative control PLA in (**A**) MCF7 and (**B**) T47D using the combination of polyclonal goat–IgG isotype with rabbit isotype antibody (left) and the combination of rabbit isotype and mouse isotype antibody (right). Cells were treated with NET, DYD, P4 ($10^{-6}$ M) or DMSO (0.01%). Nuclear stain: DAPI. Magnification 40×.

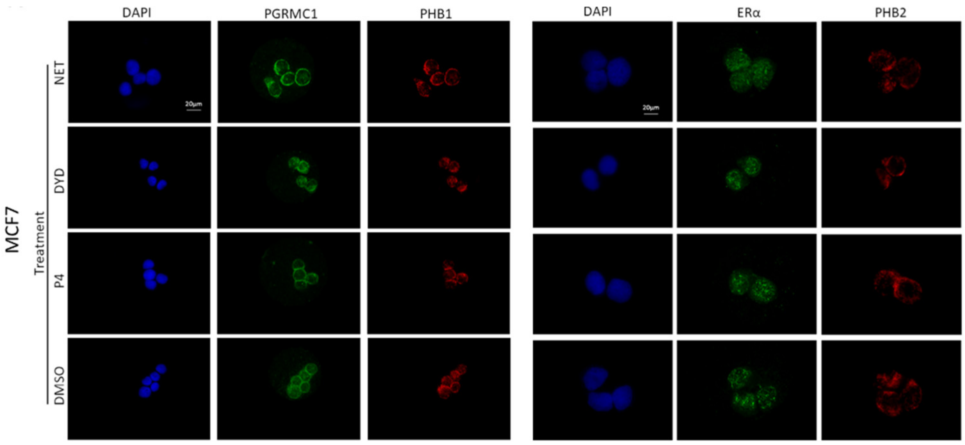


**Figure S10.** Immunofluorescence staining of MCF7 cells for PGRMC1, PHB1, ER$\alpha$ and PHB2. Cells were treated with NET, DYD, P4 ($10^{-6}$ M) or DMSO (0.01%). Nuclear stain: DAPI. Magnification 40×.

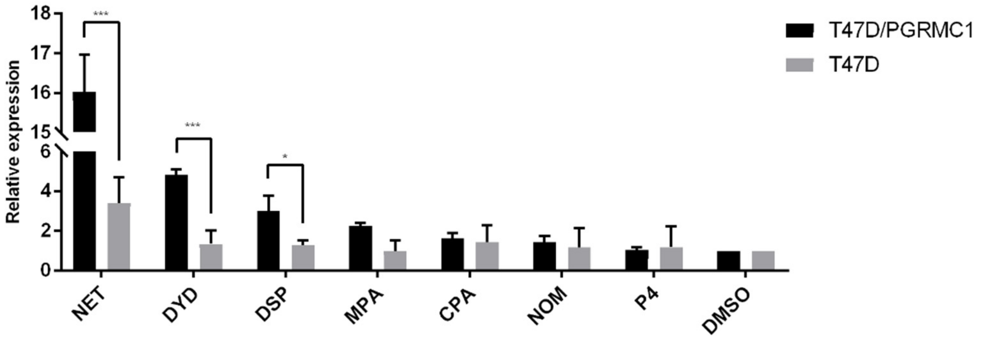


**Figure S11.** qRT−PCR analysis of *TFF1* mRNA expression in T47D/PGRMC1 and T47D cells. Cells were treated with NET, DYD, DSP, MPA, CPA, NOM, progesterone ($10^{-6}$ M) or DMSO (0.01%) for 24 h. Intensity was normalized to respective DMSO control. Statistical analysis was performed by two−way ANOVA and Bonferroni post−hoc tests. *: $p < 0.05$, ***: $p < 0.001$.

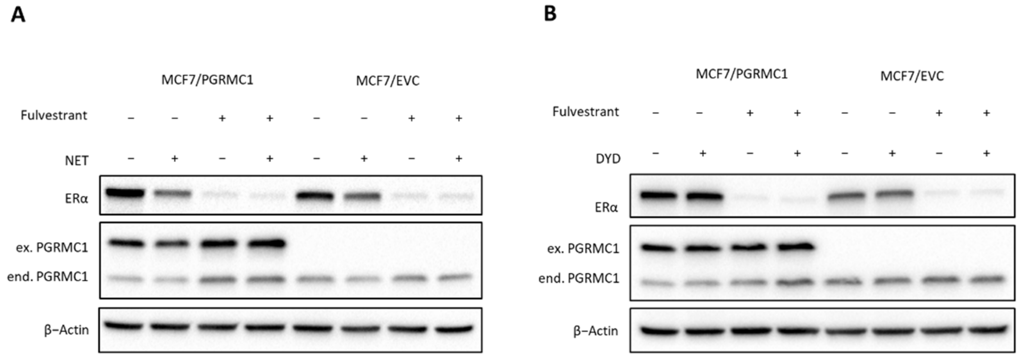


**Figure S12.** Western blot analysis of ER$\alpha$ expression after fulvestrant treatment in MCF7/PGRMC1 and MCF7/EVC cells. Cells were treated with (**A**) fulvestrant ($10^{-7}$ M) and NET ($10^{-6}$ M), (**B**) fulvestrant and DYD ($10^{-6}$ M), or DMSO, respectively.

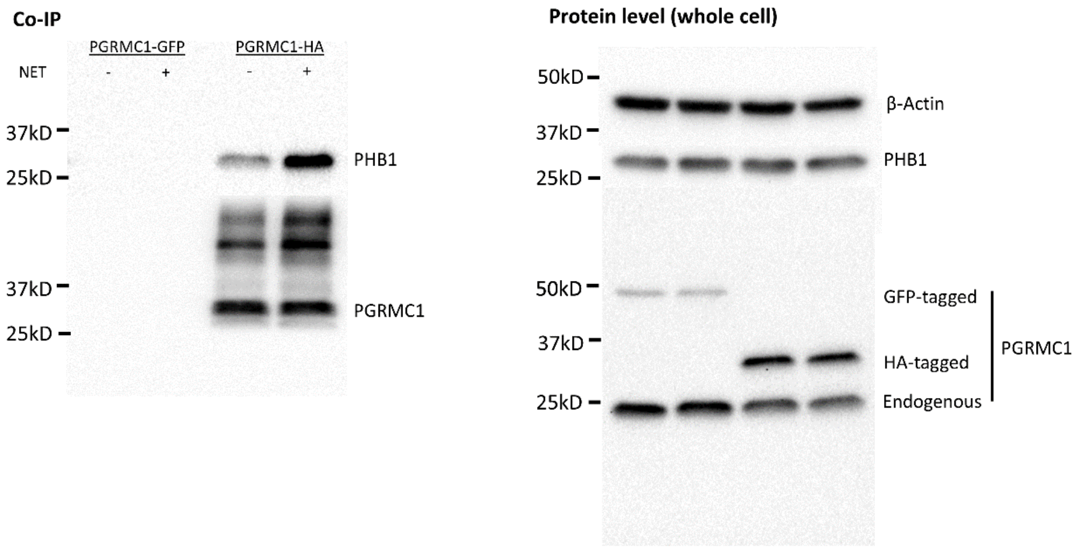


**Figure S13.** Left panel: analysis of immunopurified (HA-based) precipitate from PGRMC1 overexpressing cell lines MCF7/PGRMC1 and MCF7/PGRMC1-GFP cells; detection of PHB1 and PGRMC1. Right panel: Protein level in whole cell.

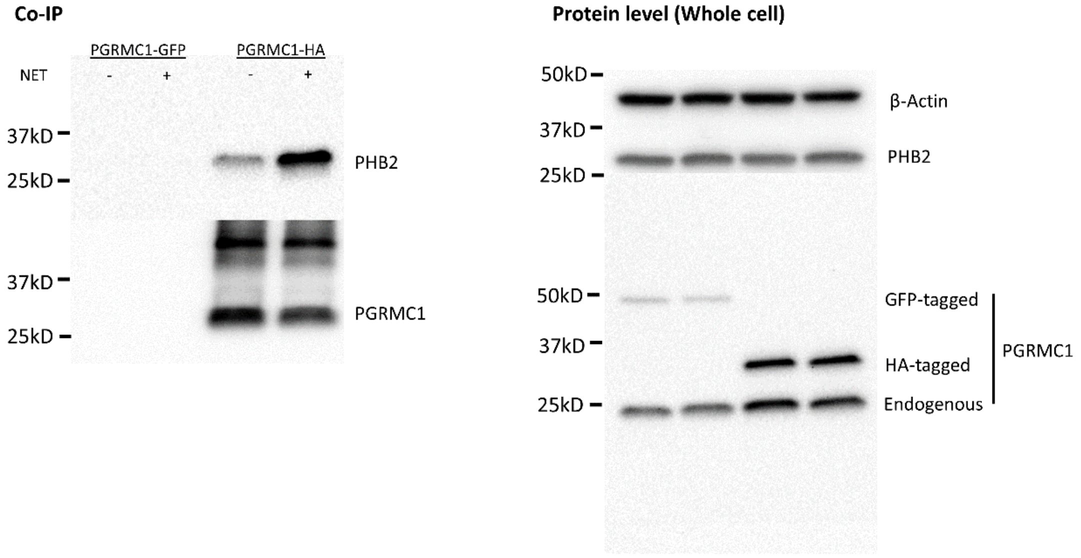


**Figure S14.** Left panel: analysis of immunopurified (HA-based) precipitate from PGRMC1 overexpressing cell lines MCF7/PGRMC1 and MCF7/PGRMC1-GFP cells; detection of PHB2 and PGRMC1. Right panel: Protein level in whole cell.

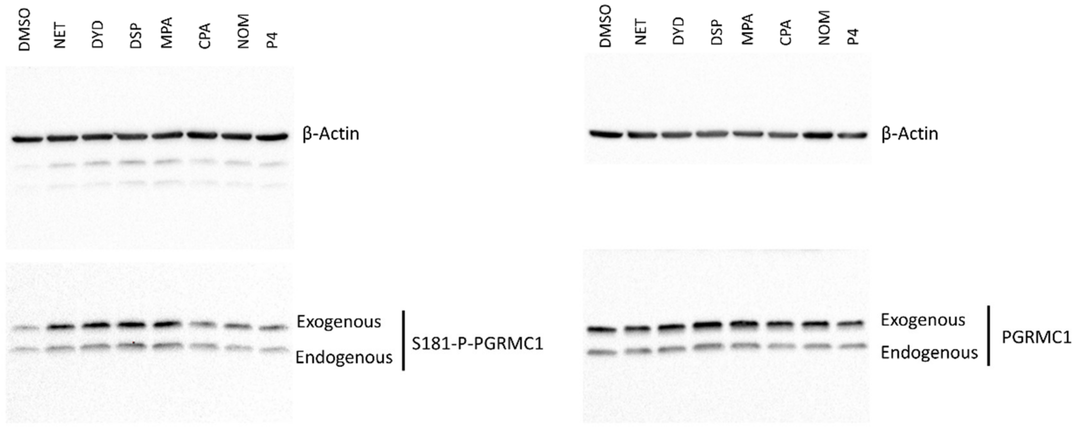


**Figure S15.** Analysis of PGRMC1-S181-phosphorylation after progestin treatment in whole cell lysates of MCF7/PGRMC1 cells. Upper panel: detection of S181-phosphorylated PGRMC1; Lower panel: detection of PGRMC1 protein level.

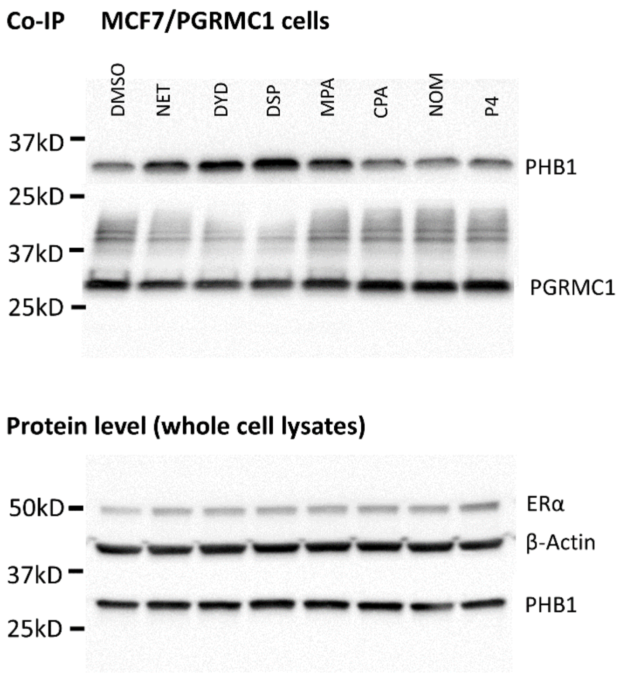


**Figure S16.** Analysis of immunopurified (HA-based) precipitate from MCF7/PGRMC1 cells after progestin treatment (upper panel) and protein level in whole cell lysates of the same samples (lower panel). Detection of PHB1.

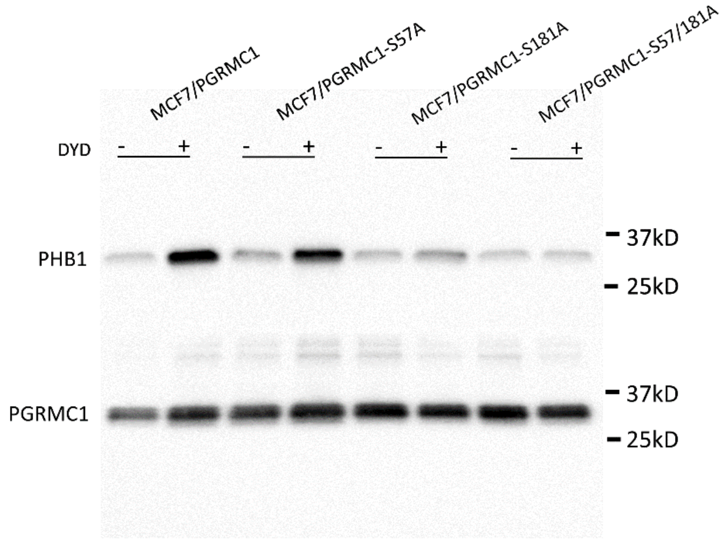


**Figure S17.** Analysis of immunopurified (HA-based) precipitate from MCF7 cells overexpressing PGRMC1 or phosphorylation-deficient PGRMC1 variants after treatment with DYD. Detection of PHB1.

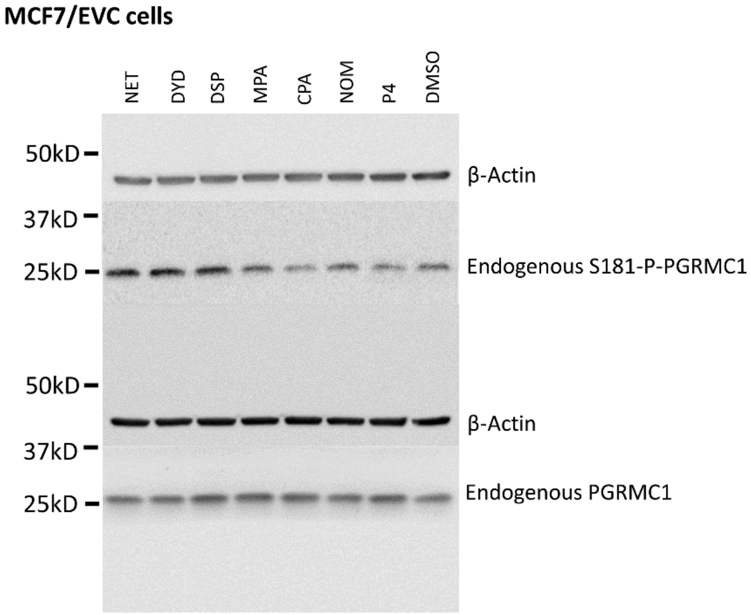


**Figure S18.** Analysis of PGRMC1-S181-phosphorylation after progestin treatment in whole cell lysates of MCF7/EVC cells. Upper panel: detection of S181-phosphorylated PGRMC1; Lower panel: detection of PGRMC1 protein level.

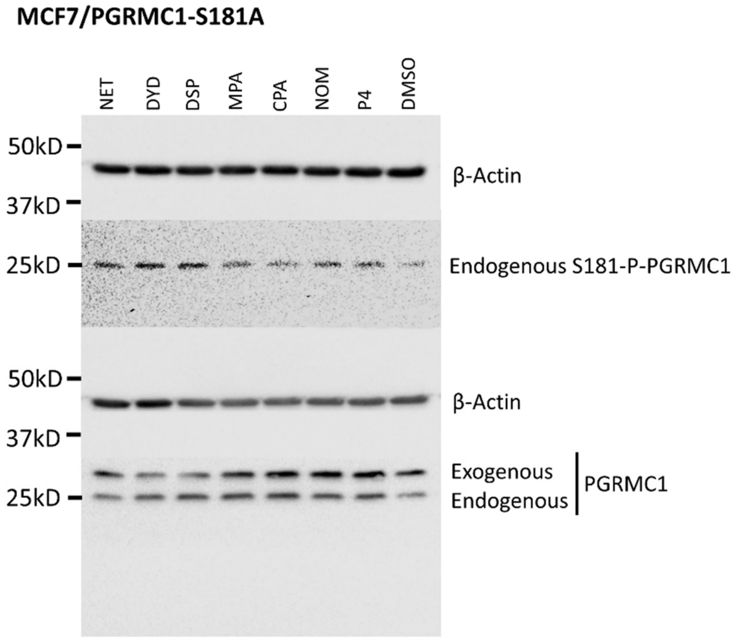


**Figure S19.** Analysis of PGRMC1-S181-phosphorylation after progestin treatment in whole cell lysates of MCF7/PGRMC1-S181A cells. Upper panel: detection of S181-phosphorylated PGRMC1; Lower panel: detection of PGRMC1 protein level.

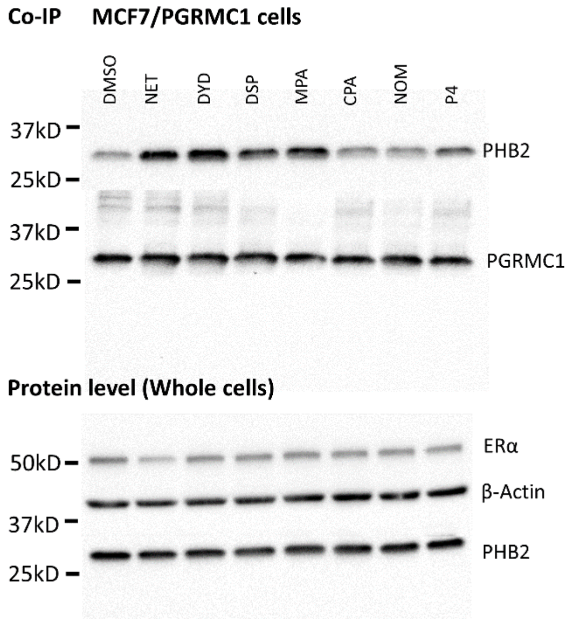


**Figure S20.** Analysis of immunopurified (HA-based) precipitate from MCF7/PGRMC1 cells after progestin treatment (upper panel) and protein level in whole cell lysates of the same samples (lower panel). Detection of PHB2.

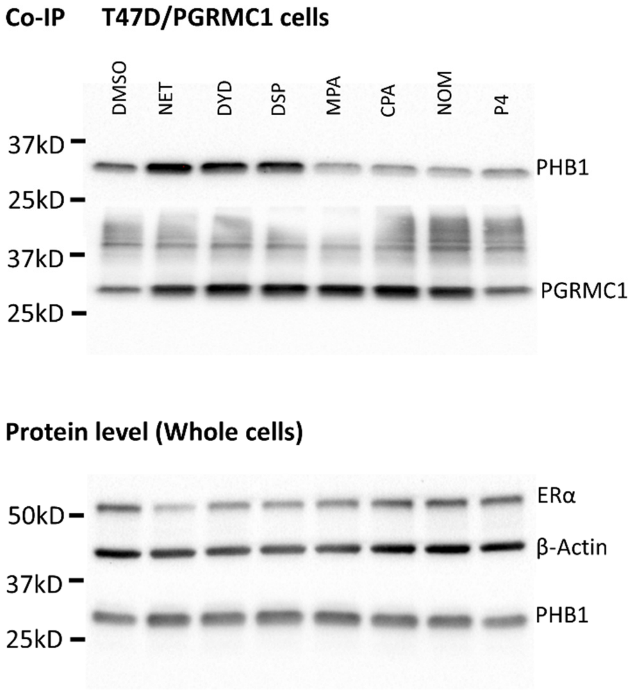


**Figure S21.** Analysis of immunopurified (HA-based) precipitate from T47D/PGRMC1 cells after progestin treatment (upper panel) and protein level in whole cell lysates of the same samples (lower panel). Detection of PHB1.

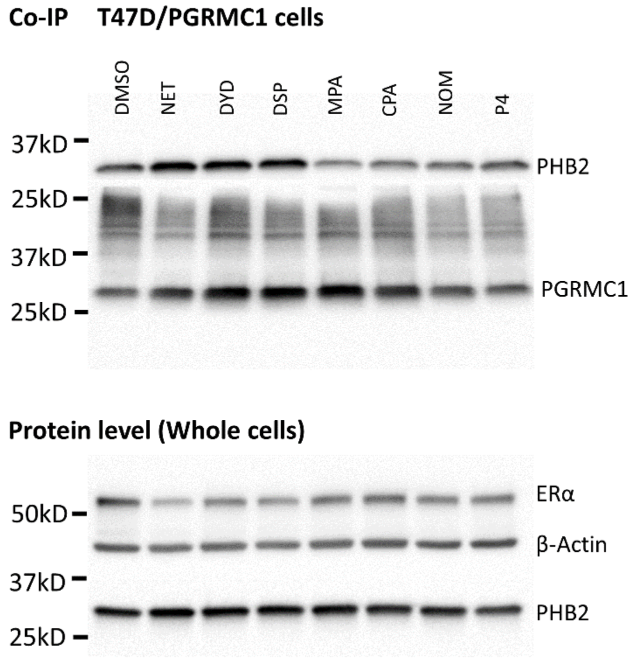


**Figure S22.** Analysis of immunopurified (HA-based) precipitate from T47D/PGRMC1 cells after progestin treatment (upper panel) and protein level in whole cell lysates of the same samples (lower panel). Detection of PHB2.

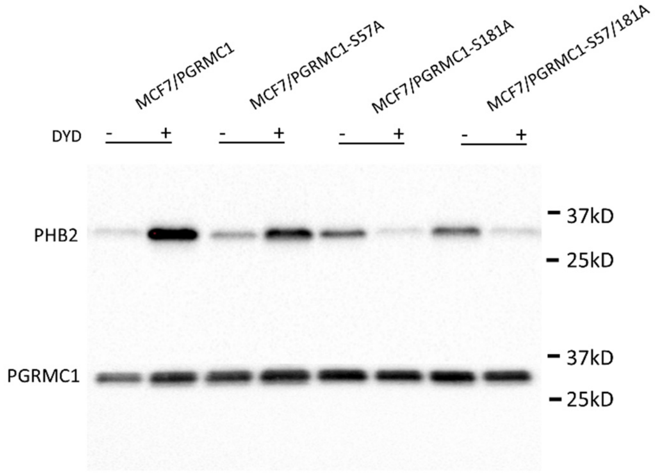


**Figure S23.** Analysis of immunopurified (HA-based) precipitate from MCF7 cells overexpressing PGRMC1 or phosphorylation-deficient PGRMC1 variants after treatment with DYD. Detection of PHB2.

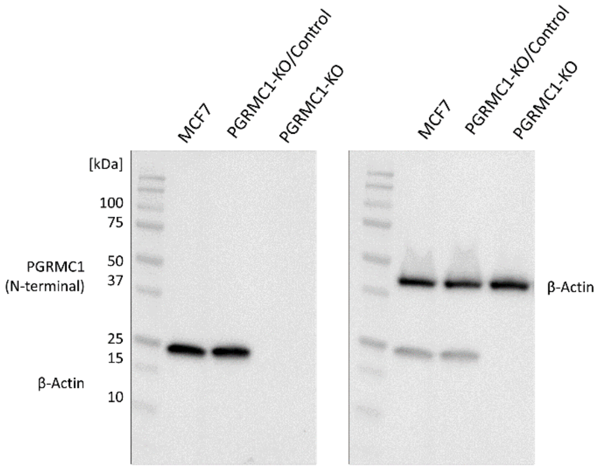


**Figure S24.** Analysis of PGRMC1-expression in whole cell lysates from MCF7, MCF7/PGRMC1-KO/Control and MCF7/PGRMC1-KO cells. Detection of PGRMC1 with the N-terminal antibody (Cell Signaling).

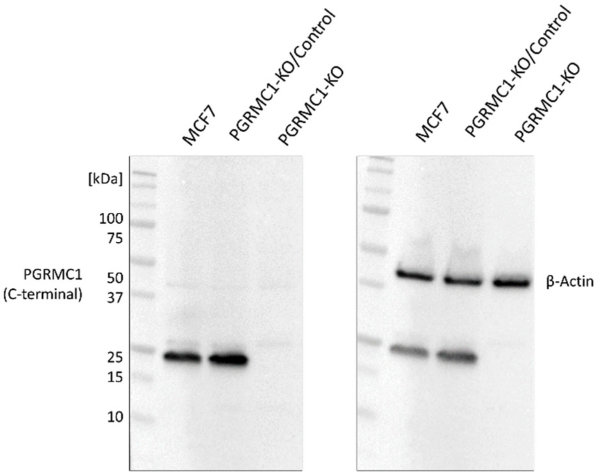


**Figure S25.** Analysis of PGRMC1-expression in whole cell lysates from MCF7, MCF7/PGRMC1-KO/Control and MCF7/PGRMC1-KO cells. Detection of PGRMC1 with the C-terminal antibody (Abcam).

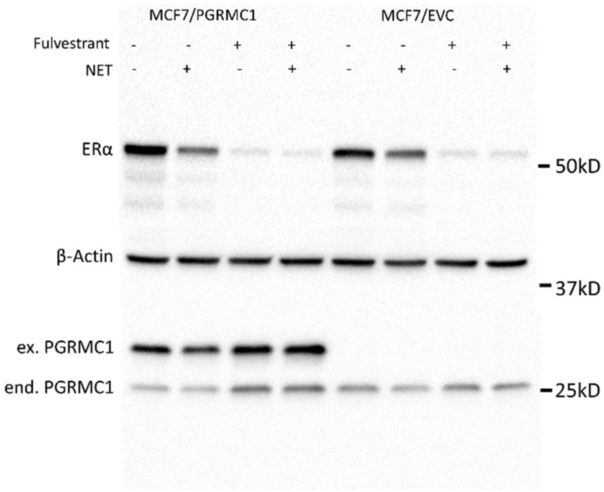


**Figure S26.** Analysis of ER$\alpha$- and PGRMC1-protein level in MCF7/PGRMC1 and MCF7/EVC cells after treatment with fulvestrant and NET.

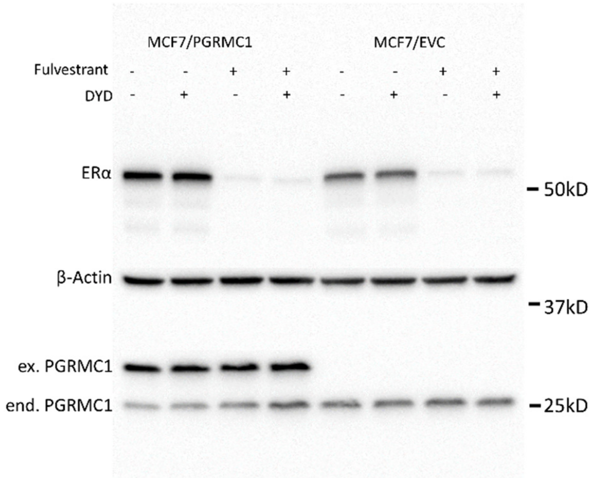


**Figure S27.** Analysis of ER$\alpha$- and PGRMC1-protein level in MCF7/PGRMC1 and MCF7/EVC cells after treatment with fulvestrant and DYD.
